# Temporal and Behaviour-Aware Multimodal Modelling for Hour-Ahead Hypoglycaemia Prediction During Ramadan Fasting in Type 1 Diabetes

**DOI:** 10.3390/s26082552

**Published:** 2026-04-21

**Authors:** Mais Alkhateeb, Rawan AlSaad, Samir Brahim Belhaouari, Sarah Aziz, Arfan Ahmed, Hamda Ali, Dabia Al-Mohanadi, Kawsar Mohamud, Najla Al-Naimi, Arwa Alsaud, Hamad Al-Sharshani, Javaid I. Sheikh, Khaled Baagar, Alaa Abd-Alrazaq

**Affiliations:** 1 College of Education and Arts, Lusail University, Lusail P.O. Box 9717, Qatar; 2 AI Center for Precision Health, Weill Cornell Medicine–Qatar, Doha P.O. Box 24144, Qatar; 3 Division of Information and Computing Technology, College of Science and Engineering, Hamad Bin Khalifa University, Doha P.O. Box 34110, Qatar; 4 Division of Diabetes and Endocrinology Qatar Metabolic Institute, Hamad Medical Corporation, Doha P.O. Box 3050, Qatar

**Keywords:** type 1 diabetes, hypoglycaemia forecasting, Ramadan fasting, continuous glucose monitoring, wearable sensors, deep learning, long short-term memory, probability calibration, class imbalance, dataset shift

## Abstract

**Highlights:**

**What are the main findings?**

- A behaviour-aware, multimodal deep learning framework enables accurate hour-ahead hypoglycaemia prediction during Ramadan fasting.
- Integration of CGM with wearable-derived physiological, behavioural, and circadian features enhances predictive performance.
- The model achieves robust and well-calibrated performance under severe real-world class imbalance (~4% events).
- Patient-wise, leak-free evaluation ensures reliable generalisation and outperforms glucose-only approaches.

**What is the implication of the main finding?**

- The framework enables proactive, clinically actionable early warning of hypoglycaemia, supporting safer fasting and improved self-management.
- Robust cross-phase generalisation between Ramadan and post-fasting periods supports real-world deployment in decision-support and wearable-integrated monitoring systems.

**Abstract:**

Ramadan fasting substantially alters meal timing, sleep patterns, and daily activity, thereby increasing the risk of hypoglycaemia in adults with type 1 diabetes (T1D). Although continuous glucose monitoring (CGM) systems provide real-time alerts, these are largely reactive or limited to short prediction horizons, offering insufficient warning under fasting-related behavioural and circadian disruption. This study aims to evaluate whether behaviour-aware, temporally enriched recurrent deep learning models, leveraging multimodal CGM and wearable-derived signals, can forecast hypoglycaemia one hour ahead during Ramadan and the post-fasting period. In an observational, free-living cohort study conducted in Qatar, 33 adults with T1D were monitored using CGM and a wrist-worn wearable during Ramadan 2023 and the subsequent month. Multimodal data were aggregated into hourly features and organised into rolling 36 h sequences. In addition to physiological signals, explicit temporal and circadian proxy features were engineered, including cyclic time encodings, day–night indicators, and Ramadan-specific behavioural windows (e.g., pre-iftar, iftar, post-iftar, and fasting phases). Recurrent models, including LSTM and BiLSTM architectures, were trained using patient-wise, leak-free splits, with focal loss applied to address class imbalance. Model performance was evaluated on a held-out, naturally imbalanced test set using ROC AUC, precision–recall AUC, recall, and probability calibration, alongside cross-phase evaluation between Ramadan and post-fasting periods. Following quality control, 1164 participant-days were retained, with hypoglycaemia accounting for approximately 4% of hourly observations. Temporal feature enrichment and the use of a 36 h lookback window improved both discrimination and calibration, with performance stabilizing beyond this horizon. On the imbalanced test set, the best-performing multimodal model achieved an ROC AUC of 0.867 and a precision–recall AUC of 0.341, identifying 77% of next-hour hypoglycaemic events at a sensitivity-focused operating point (precision = 0.14). The selected BiLSTM model demonstrated good probability calibration (Brier score ≈ 0.03). Models trained using wearable-derived inputs alone achieved comparable discrimination and, in some configurations, higher precision–recall AUC than CGM-only baselines. Notably, models trained on the original imbalanced data outperformed resampled variants, suggesting that temporal and behavioural features provided sufficient discriminatory signal without requiring aggressive class balancing. Cross-phase evaluation indicated robust generalisation, particularly for the BiLSTM model. Overall, behaviour-aware, temporally enriched multimodal models can provide calibrated, hour-ahead hypoglycaemia risk estimates during Ramadan fasting in adults with T1D, enabling proactive intervention beyond reactive CGM alerts. Explicit modelling of circadian and behavioural dynamics enhances predictive performance under real-world class imbalance. Furthermore, integrating wearable-derived behavioural and physiological signals adds predictive value beyond CGM alone, supporting robustness across varying levels of contextual data availability. External validation and prospective clinical evaluation are required prior to deployment.

## 1. Introduction

### 1.1. Background and Prior Work

Ramadan is observed annually by Muslims worldwide, during which healthy adults abstain from food and drink from dawn until sunset. For adults living with type 1 diabetes mellitus (T1DM), fasting introduces interrelated physiological and behavioural challenges that can compromise glycaemic stability. Prolonged fasting, heavier evening meals at iftar, late-night snacking, reduced daytime activity, and disrupted or fragmented sleep reshape circadian rhythms and glucose metabolism [1]. Even with advanced hybrid closed-loop (AHCL) systems, these Ramadan-specific changes may destabilise glucose control and increase vulnerability to hypoglycaemia [2].

Hypoglycaemia, defined as blood glucose ≤ 70 mg/dL (3.9 mmol/L), remains a central barrier to safe insulin therapy. Population-based studies indicate that 30–40% of adults with T1DM experience at least one severe hypoglycaemic event annually [3,4,5]. Such episodes produce autonomic and neuroglycopenic manifestations—including tremor, sweating, tachycardia, irritability, impaired attention, and visual disturbance—and, when prolonged or recurrent, may progress to seizures, arrhythmias, coma, or death [6,7,8,9]. These Ramadan-specific behavioural shifts further amplify hypoglycaemia risk, reinforcing the need for reliable early-warning systems to support safer fasting [2].

Continuous glucose monitoring (CGM) has transformed diabetes management by providing near-continuous insight into glycaemic dynamics and reducing hypoglycaemia incidence. However, most current CGM alerts remain threshold-based or rely on simple rate-of-change rules, which are largely reactive and may provide limited lead time under fasting-related behavioural and circadian disruption [1,10]. In contrast to reactive alarms that signal imminent or ongoing hypoglycaemia, forecasting aims to anticipate risk before glucose thresholds are crossed, enabling preventive rather than corrective action.

Moreover, CGM signals alone do not capture broader physiological and behavioural determinants of glycaemic variability, such as heart-rate dynamics, sleep architecture, physical activity, hydration status, and autonomic responses [11]. Wearable technologies provide continuous, non-invasive monitoring of activity, heart rate, sleep stages, and energy expenditure. When integrated with CGM, these multimodal streams offer a richer representation of metabolic context and of the physiological states that precede hypoglycaemic events, particularly during Ramadan when behavioural rhythms differ substantially from non-fasting periods [12,13].

However, signals derived from consumer-grade wearable devices must be interpreted with appropriate caution. Prior validation studies have shown that wrist-worn sensors provide reasonably accurate estimates of heart rate and energy expenditure. Accordingly, wearable-derived measures are treated here as contextual indicators rather than clinical-grade physiological measurements [14].

Previous research on hypoglycaemia prediction has largely focused on CGM-based models developed for narrow physiological contexts, including nocturnal hypoglycaemia [15,16] and postprandial risk using supervised learning approaches [17,18]. Other high-performing models have been developed for inpatient environments or electronic health record data [19,20], which are not available in free-living settings such as Ramadan fasting.

Wearable-based studies have primarily targeted hypoglycaemia detection rather than forward prediction [21]. More recent multimodal and explainable machine-learning frameworks have emerged [22,23], but these have been developed in non-fasting populations and rarely evaluated probability calibration, robustness, or sleep-stage physiology.

Ramadan-specific predictive research remains limited and has focused almost exclusively on adults with type 2 diabetes. The PROFASTIT study examined glucose variability during fasting [24] but did not address hypoglycaemia prediction and did not include individuals with type 1 diabetes. Established expert guidance, including the IDF–Diabetes and Ramadan (IDF-DaR) Practical Guidelines, emphasises structured risk stratification, patient education, and proactive glucose monitoring for people with diabetes who choose to fast during Ramadan [2]. Despite these recommendations, no published studies have combined CGM and wearable-derived physiological signals to provide hour-ahead hypoglycaemia prediction in fasting adults with type 1 diabetes.

Beyond wearable-derived physiological signals, environmental factors such as ambient light exposure are increasingly recognised as key modulators of circadian regulation and metabolic processes. Light acts as the primary external zeitgeber of the human circadian system, influencing hormonal secretion, sleep–wake cycles, and glucose metabolism. Recent evidence indicates that both the timing and intensity of light exposure significantly affect glycaemic control. Controlled experimental studies have demonstrated that exposure to natural daylight improves glucose regulation and increases time spent within the normoglycaemic range [25]. Conversely, large-scale prospective analyses using wearable light sensors have shown that circadian-disruptive light patterns, including artificial light at night, are associated with increased metabolic dysfunction and type 2 diabetes risk [26]. These effects are thought to be mediated through circadian misalignment, melatonin suppression, and downstream metabolic pathways [27].

Ramadan therefore represents a predictable yet substantial covariate shift in sleep, meals, activity, and circadian organisation, making it a natural stress-test for the robustness of real-world prediction models. More details on prior hypoglycaemia prediction studies are summarised in Table A1.

### 1.2. Research Problem and Study Aims

To address these gaps, this study develops a temporal and behaviour-aware, multimodal deep-learning framework that forecasts hypoglycaemia one hour in advance for free-living adults with T1DM during Ramadan. Rather than treating fasting as a generic time period and relying primarily on glucose trajectories, our approach fuses high-resolution CGM with wearable-derived signals spanning activity, cardiorespiratory physiology, and sleep. This enables the model to capture fasting-related behavioural shifts and circadian disruption that materially shape short-horizon risk.

A one-hour prediction horizon is clinically meaningful: it is long enough to permit preventive action (e.g., carbohydrate intake, insulin adjustment, or activity modification), yet short enough to maintain forecasting accuracy under rapidly changing physiological conditions. Methodologically, we prioritise clinical credibility through patient-wise, leak-free evaluation and explicit reporting of probability calibration, which is essential when predicted risks may inform safety-critical decisions. We further evaluate cross-phase robustness by testing generalisability between Ramadan and the post-fasting period, reflecting real deployment scenarios in which physiology and behavioural routines shift while hypoglycaemia remains rare.

We hypothesise that multimodal models will outperform CGM-only approaches and that recurrent architectures will retain discriminative performance and calibration across Ramadan and post-fasting distribution shifts. This evaluation goes beyond ranking risk and instead assesses whether models can produce reliable, calibrated risk estimates under natural class imbalance. Overall, this work advances hour-ahead hypoglycaemia forecasting in Ramadan from proof-of-concept toward deployable, personalised risk monitoring that supports safer fasting and day-to-day self-management. Despite this growing body of evidence, ambient light exposure remains underutilised in wearable-based glucose prediction models, largely due to the limited availability of reliable light-sensing data in real-world clinical datasets. In this context, temporal features and behaviourally informed proxy variables provide a practical alternative for capturing circadian structure. This is particularly relevant during Ramadan, where daily routines are substantially altered, including shifts in meal timing, sleep patterns, and nocturnal activity.

Beyond static temporal encoding, the present study explicitly models circadian phase transitions and fasting–feeding state dynamics using time-relative features (e.g., hours to/from key events such as iftar and suhoor) and cyclic representations. This enables the model to capture non-stationary physiological responses across the day, particularly during transition windows associated with rapid glycaemic fluctuations.

Accordingly, the proposed framework integrates multimodal physiological signals with temporal encodings and Ramadan-specific behavioural proxy features to approximate circadian dynamics and enhance hypoglycaemia risk prediction.

In summary, the key contributions of this work are:

- A temporal and behaviour-aware, multimodal deep-learning framework for hour-ahead hypoglycaemia forecasting in free-living adults with type 1 diabetes during Ramadan.
- Explicit modelling of circadian phase transitions and fasting–feeding dynamics using time-relative and cyclic temporal features, enabling the capture of non-stationary glycaemic risk patterns during critical transition windows (e.g., pre-iftar and postprandial periods).
- Multistream fusion of CGM with wearable-derived activity, cardiorespiratory, and sleep signals to represent fasting-specific behavioural and physiological context.
- Patient-wise, leak-free evaluation under severe real-world class imbalance, using rare-event-appropriate metrics (including precision–recall analysis).
- Systematic calibration reporting and cross-phase generalisability testing between Ramadan and the post-fasting period to assess reliability under physiological and behavioural shifts.
- Demonstration that wearable recurrent models can approach CGM-based performance for short-horizon hypoglycaemia risk estimation, supporting graceful degradation when glucose data are limited.

This work is motivated by the clinical challenge of maintaining glycaemic stability during intentional dietary restriction, such as Ramadan fasting, where hypoglycaemia risk is increased due to prolonged fasting intervals and altered circadian behaviour. By integrating multimodal physiological and behavioural data, the proposed framework aims to support clinically meaningful, context-aware hypoglycaemia prediction.

The remainder of this article is structured as follows. Section 2 describes the study design, participant cohort, multimodal data sources, preprocessing pipeline, and modelling framework, including strategies for handling severe class imbalance and ensuring leak-free evaluation. Section 3 presents the results, including cohort characteristics, model discrimination and calibration under natural and balanced prevalence, robustness across fasting and post-fasting phases, and patient-level explainability analyses. Section 4 discusses the principal findings in the context of prior work, methodological strengths and limitations, and implications for clinical deployment and future research. Finally, Section 5 concludes by summarising the contribution of behaviour-aware, hour-ahead hypoglycaemia forecasting and outlining next steps toward prospective evaluation and real-world decision support.

## 2. Methods

### 2.1. Study Design and Participants

**Figure 1:**
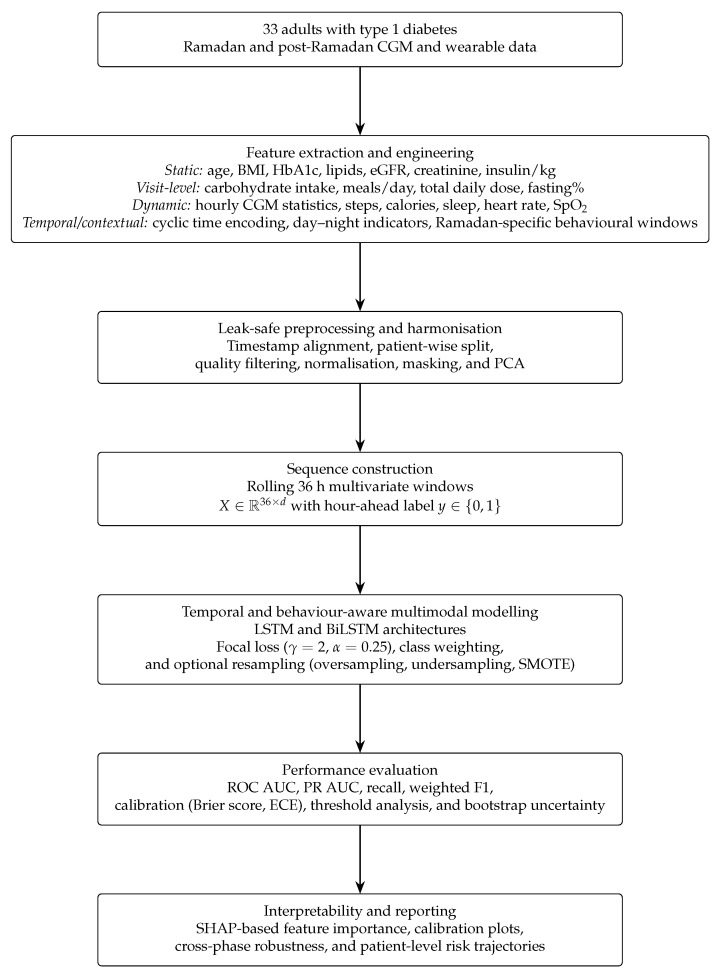
Pipeline for temporal and behaviour-aware hour-ahead hypoglycaemia forecasting. Multimodal data from 33 adults with type 1 diabetes were organised into static, visit-level, dynamic CGM–wearable, and temporal/contextual feature blocks, including circadian proxy variables and Ramadan-specific behavioural windows. After leak-safe preprocessing, harmonisation, and dimensionality reduction, the data were assembled into rolling 36 h sequences and analysed using recurrent deep-learning models. Model outputs were evaluated for discrimination, calibration, threshold robustness, and cross-phase generalisability, with interpretability provided through SHAP-based feature attribution and patient-level risk trajectories.

We conducted an observational cohort study of adults with type 1 diabetes mellitus (T1DM) during Ramadan 2023 and the subsequent non-fasting month in Qatar. Participants were recruited consecutively from the Division of Diabetes and Endocrinology at Hamad Medical Corporation as part of routine specialist follow-up for Ramadan fasting. Thirty-five individuals consented to participate; two were excluded after quality control because of insufficient usable CGM–wearable overlap, leaving 33 participants with analysable intraday sequences (Table A2). Ramadan spanned 22 March–19 April 2023, with follow-up extending to 19 May 2023. A schematic overview of the data flow and modelling pipeline is shown in Figure 1.

Eligible participants were adults aged 18 years or older with established T1DM, treated with a Medtronic MiniMed 780G (Medtronic plc, Dublin, Ireland) advanced hybrid closed-loop (AHCL) system and intending to fast during Ramadan under specialist supervision. Exclusion criteria included pregnancy, advanced renal impairment, recent diabetic ketoacidosis, or any condition judged by the treating diabetologist to preclude safe fasting. Seven structured clinic visits—from pre-Ramadan through the late post-fasting month—captured anthropometric, biochemical, behavioural, and device-use information. Site codes (R01–R43) were standardised and linked to unique patient identifiers, visit indices (1–7), and fasting-phase categories (pre-fasting, Ramadan, and after-fasting), as detailed in Appendix B.1 and Table A3.

No formal sample size calculation was performed; all eligible individuals who consented and provided sufficient sensor coverage were included. The resulting cohort represents a controlled real-world setting of adults with T1DM using a single advanced hybrid closed-loop system within one healthcare network, allowing focused evaluation of multimodal hypoglycaemia forecasting under fasting conditions.

All study procedures adhered to the Declaration of Helsinki and were approved by the Hamad Medical Corporation Institutional Review Board and Medical Research Center (IRB/MRC). Written informed consent was obtained from all participants prior to enrolment, and all datasets were fully de-identified and stored on secure, access-restricted servers within the HMC research environment.

Table 1 summarises the cohort’s clinical and physiological characteristics, including baseline metabolic markers and the yield of CGM and wearable data. The table also illustrates data completeness, showing consistently high CGM coverage and greater variability across wearable modalities—patterns that informed subsequent preprocessing and model-design decisions.

### 2.2. Procedures

Each participant used a Medtronic MiniMed 780G advanced hybrid closed-loop (AHCL) system, which recorded interstitial glucose at 5-min intervals and automatically adjusted basal insulin delivery with integrated safety alarms. In parallel, participants wore a Huawei Band 6, which captured minute-level wearable signals including step count, heart rate, calorie expenditure, peripheral oxygen saturation (SpO_2_), and sleep-stage estimates. Wearable-derived sleep stages and SpO_2_ were treated as device-reported indices for contextual modelling rather than clinical-grade physiological measurements.

The PROFAST data-engineering framework (an internal pipeline developed for prospective fasting studies) was adapted to harmonise high-frequency CGM and wearable data streams (Table A4). The pipeline was implemented in Python using reproducible, version-controlled scripts (Appendix B). CGM coverage remained consistently high (exceeding 80% of expected samples after filtering), whereas wearable data completeness varied by modality, typically ranging between 50% and 70%, primarily due to intermittent off-wrist periods.

All participants were monitored using a standardised device ecosystem comprising the Medtronic 780G continuous glucose monitoring system and Huawei Band 6 wearable sensors. This unified configuration ensured consistency in data acquisition and minimised inter-device variability; while this controlled setup strengthens internal validity, it may limit generalisability to alternative device platforms and more heterogeneous real-world settings. The study design and data acquisition strategy were selected to support internal consistency and transparent reporting of data sources and potential sources of bias.

All time-series data were aligned to ISO-standard start-of-hour timestamps to enable multimodal integration. Glucose values were converted to mg/dL (1 mmol/L = 18.0182 mg/dL) to ensure consistency with clinical thresholds. A calendar day was retained only if at least 50% of expected CGM readings were available, and hourly summaries excluded hours with fewer than four valid CGM measurements to ensure reliable aggregation. Details of quality control and missingness handling are provided in Appendix B.

No temporal imputation methods (e.g., forward filling, interpolation, or mean imputation across time) were applied to reconstruct missing wearable or CGM values. Instead, data quality was ensured through filtering criteria, including exclusion of hours with insufficient CGM observations (fewer than four readings per hour). CGM features were derived using within-hour aggregation statistics (e.g., mean and standard deviation) computed from available measurements. This aggregation reflects feature extraction rather than imputation, as no missing values were artificially reconstructed. Missing wearable values were retained and handled using channel-wise masking indicators, allowing the model to learn informative patterns of missingness without introducing artificial temporal structure.

Hourly CGM statistics (minimum, maximum, mean, standard deviation, and composite cgm_mean±cgm_std) and wearable signals (steps, energy expenditure, distance, heart rate, SpO_2_, and sleep-stage durations) were aggregated onto a common hourly grid to ensure temporal alignment across modalities. PCA, fitted exclusively on the training data, was used to derive compact latent representations of CGM and wearable feature blocks. For CGM features, three components explained 74.2%, 25.7%, and 0.1% of variance, respectively. For wearable-derived lifestyle features, three components explained 88.3%, 9.0%, and 1.7% of variance. Although the third component contributed minimally to total explained variance, it was retained initially to ensure consistent dimensionality across feature modalities and to preserve interpretability of the three-domain structure (activity/energy, physiology, and sleep/rest). Its necessity was subsequently evaluated through sensitivity analyses in which models were re-trained after excluding the third PCA component from each feature block.

Static baseline covariates (age, BMI, HbA1c, lipid profile, eGFR, creatinine, blood pressure, SmartGuard%) captured cardiometabolic status, renal function, and device-use behaviour. Ramadan-phase visit-level variables (Visits 2–5) were organised into three domains: (i) glycaemic indices (time-in-range, total time <70 mg/dL, glucose variability, CV%, GRI); (ii) insulin/device parameters (total daily dose, bolus%, auto-basal%, auto-correction%); and (iii) lifestyle factors (meals/day, carbohydrate intake, fasting%, fasting days), with additional metadata (hour_of_day, visit_assigned, period_main) supporting circadian and phase-specific interpretation. The complete feature set was structured into dynamic, visit-level, static baseline, contextual, and outcome components, as detailed in Table A5 and Table A6.

Ambient light exposure data were not available in the present dataset and were therefore not included as model inputs. Temporal features (e.g., hour-of-day encoding) and Ramadan-specific phase indicators (e.g., fasting, pre-iftar, and post-iftar periods) were instead used to capture circadian and behavioural structure. For clarity, the variable *light* in the wearable dataset refers to sleep-stage classification (i.e., light sleep) rather than environmental light exposure.

To investigate the role of temporal structure in hypoglycaemia prediction during Ramadan, an extended feature-engineering strategy was introduced. In addition to the original multimodal inputs, explicit temporal and circadian proxy variables were constructed, including cyclical time encoding (*hour_sin*, *hour_cos*), day–night indicators, Ramadan-specific behavioural windows (pre-iftar, iftar, post-iftar, suhoor, and late-night periods), and feeding–fasting phase indicators. Short-term CGM dynamics were incorporated through first- and second-order differences, rolling statistics, slope estimates, and time since the last hypoglycaemic event. These features were integrated with CGM summary statistics, PCA-derived components, static variables, and visit-level information to form a comprehensive multimodal representation. All preprocessing steps, including scaling and feature integration, were applied consistently across experiments.

### 2.3. Model Input Scenarios

To clarify how different data streams were utilised, we evaluated multiple predefined model-input scenarios throughout this study. In all configurations, CGM-derived time-series features were retained as the core glycaemic signal, and additional feature groups were incorporated to assess their incremental contribution:

- Multimodal (all features): CGM time-series features combined with wearable-derived activity, cardiovascular, and sleep signals, together with visit-level and static clinical covariates.
- CGM-only: CGM-derived time-series features without additional wearable or contextual inputs.
- CGM + wearable: CGM time-series features combined with wearable-derived activity, cardiovascular, and sleep signals.
- CGM + wearable + static: CGM and wearable features combined with static baseline clinical variables.
- CGM + wearable + static + visit-level: CGM and wearable features combined with static and visit-level clinical variables.
- CGM + wearable + static + temporal: CGM and wearable features combined with static variables and temporal/circadian features, including Ramadan-specific behavioural proxies.

These scenarios were designed to evaluate the incremental value of multimodal integration and the contribution of behavioural, clinical, and temporal context to hypoglycaemia prediction, while maintaining CGM as the primary physiological reference signal across all configurations. Model selection and configuration were guided by empirical evaluation on clinically derived data rather than purely theoretical considerations, ensuring that the final architecture reflects real-world physiological patterns and predictive performance in the target population.

### 2.4. Data Preprocessing and Split

All preprocessing steps, including feature scaling, dimensionality reduction via principal component analysis (PCA), and handling of missingness, were fitted exclusively on the training subset and applied unchanged to the validation and test subsets. Continuous features were standardised using z-score normalisation based on training-set statistics.

After quality control and filtering based on CGM data completeness, approximately 80% of the originally available hourly records were retained for analysis. This filtering step reflects data availability and quality constraints and is distinct from model training and evaluation splits.

For model development, the filtered dataset was partitioned at the participant level into training (70%), validation (10%), and testing (20%) subsets. This patient-wise splitting strategy ensured leak-free evaluation on previously unseen individuals, such that all sequences from a given participant were assigned exclusively to a single subset, thereby preventing temporal and subject-level information leakage.

Hours with fewer than four valid CGM readings were excluded, and CGM and wearable time series were aligned to a common hourly grid. Full details of preprocessing, harmonisation, and quality-control procedures are provided in Appendix B.

Harmonised hourly records were transformed into rolling 36 h sequences with a stride of one hour (see Section 2.6). Each participant contributed multiple overlapping sequences; however, all sequences from the same individual were confined to a single subset.

Because overlapping sequences introduce within-participant correlation, statistical uncertainty was quantified using participant-level bootstrap resampling, as described in the Section 2.7.

Wearable data completeness ranged between 50 and 70% across modalities, primarily due to intermittent off-wrist behaviour, including device removal during sleep, charging periods, and other user-related factors. Missingness was therefore considered partially systematic rather than purely random, with greater prevalence in nocturnal and sleep-related signals.

To preserve physiological integrity and avoid introducing artificial temporal structure, no imputation strategies were applied. Instead, missing values were retained and handled using channel-wise masking indicators, allowing the model to learn informative patterns of missingness without hallucinating data. This approach aligns with TRIPOD-AI recommendations by explicitly characterising missingness patterns and avoiding imputation strategies that may introduce bias in time-series modelling.

### 2.5. Deep-Learning Models and Imbalance Handling

We evaluated two main model families: (i) classical baselines (logistic regression, Random Forest, and XGBoost) trained on aggregated non-sequential features, and (ii) recurrent neural network architectures designed to capture temporal glucose dynamics. The sequential family included LSTM, BiLSTM, and regularised LSTM_25_ (L1/L2) variants. All recurrent models comprised two stacked LSTM-based layers, followed by a ReLU dense projection and a sigmoid output estimating next-hour hypoglycaemia risk from the preceding 36 h window. More information about the deep learning pipeline in Appendix D and the structure of recurrent neural network architectures can be found in Table A7.

In the multimodal setting, dual-input networks combined a temporal CGM (and wearable) branch with a compact static branch (32 → 25 units), with learned embeddings concatenated immediately before the final sigmoid layer. Models were trained for up to 100 epochs using early stopping based on validation PR AUC, with dropout and L2 regularisation applied to mitigate overfitting. All experiments used fixed random seeds to ensure reproducibility.

Balanced test set evaluation was included to provide an interpretable assessment of model performance under equal class distribution, complementing evaluation on the naturally imbalanced dataset and ensuring robust interpretation of sensitivity and precision.

Given the natural class imbalance of approximately 1:23 (about 4% hypoglycaemic hours), models were trained using class-weighted focal loss (α=0.25, γ=2.0) and optimised with the Adam optimiser (learning rate $10^{-3}$, batch size 64). Three training distributions were evaluated: (i) the original imbalanced dataset, (ii) a balanced diagnostic dataset created via sequence-level oversampling, and (iii) hybrid resampling strategies using undersampling, simple oversampling, or SMOTE-based methods. Static covariates were never synthesised. SMOTE-based variants were explored as diagnostic sensitivity analyses because synthetic time-series sequences may not preserve physiological realism. Model selection was performed on the validation set using a combination of precision–recall AUC and calibration metrics (Brier score and expected calibration error). The best performed architecture and hyperparameters are reported in Table A8.

### 2.6. Outcomes

For descriptive purposes only, a calendar day was classified as a hypoglycaemia day if any CGM value was ≤70 mg/dL (3.9 mmol/L). After quality filtering, the final dataset comprised 1164 labelled days (208 hypoglycaemic and 956 non-hypoglycaemic) and approximately 19,000 hourly windows derived from 60 min intervals with at least four valid CGM readings.

The primary modelling outcome was defined at the hourly level as hour-ahead hypoglycaemia, corresponding to the occurrence of any CGM value ≤70 mg/dL (3.9 mmol/L) in the subsequent hour (hypo_label = 1); all other hours were labelled 0. A one-hour prediction horizon was selected as a clinically actionable interval, providing sufficient time for carbohydrate intake, insulin adjustment, or modification of imminent activity. Secondary outcomes focused on model performance, calibration, robustness, and alert stability.

Harmonised hourly records were transformed into rolling multivariate sequences using a fixed lookback window of 36 h and a stride of one hour, with each sequence labelled by the occurrence of hypoglycaemia in the subsequent hour. The choice of the 36 h lookback window was guided by both clinical reasoning and empirical evaluation. From a physiological perspective, this window captures short-term glycaemic dynamics alongside broader circadian and behavioural patterns specific to Ramadan fasting, including the fasting-to-iftar cycle, delayed effects of sleep disruption, insulin dosing adjustments, and carryover effects from the preceding day.

From a modelling perspective, the 36 h window provides a balance between sufficient temporal context and computational tractability for short-horizon forecasting. Shorter windows (e.g., 6–24 h) may fail to capture delayed behavioural and metabolic effects, whereas longer windows increase computational complexity without providing clear performance benefits.

The final dataset comprised 18,499 hourly windows derived from rolling 36 h sequences (Table 2). Following patient-wise partitioning, these sequences were allocated to the training (12,870 sequences), validation (1991 sequences), and testing (3638 sequences) subsets.

All sequences from a given participant were confined to a single subset to ensure leak-free evaluation and unbiased assessment of model generalisation.

Hypoglycaemia events were relatively rare across all subsets, reflecting the inherent class imbalance of real-world CGM data. Specifically, the training set contained 604 hypoglycaemic and 12,266 non-hypoglycaemic sequences, the validation set contained 80 and 1911 sequences, and the test set contained 133 and 3505 sequences, respectively.

### 2.7. Performance Analysis

Performance was evaluated on a held-out, patient-independent test set to emulate deployment to previously unseen individuals. Given the pronounced class imbalance (approximately 4% hypoglycaemic hours), evaluation followed recommended practice for rare-event biomedical prediction [28,29].

#### 2.7.1. Baseline Alert Comparator

To contextualise model performance against common clinical practice, we compared recurrent models against a simple rule-based CGM alert baseline. This comparator flagged hypoglycaemia risk when either (i) the current CGM value fell below 70 mg/dL or (ii) the projected linear rate of glucose decline crossed the 70 mg/dL threshold within the next hour. The baseline was evaluated on the same held-out test data using identical metrics, providing a reference for the incremental value of learned temporal models over threshold- and trend-based alerts (Appendix E).

#### 2.7.2. Discrimination, Calibration, and Alert Burden

Discriminative performance was quantified using the area under the receiver-operating characteristic curve (ROC AUC) and, more importantly, the area under the precision–recall curve (PR AUC), which better reflects minority-class performance under severe imbalance [28]. Recall (sensitivity) for the hypoglycaemia class was reported across operating thresholds, with particular attention to sensitivity-oriented settings that balance event capture against false-alert burden [30]. Weighted F1 score and overall accuracy were reported for completeness but interpreted cautiously because of dominance by the majority class.

In this study, we distinguish between a classification threshold and an operating threshold. The classification threshold refers to a general probability cut-off used to convert predicted probabilities into binary labels (hypoglycaemia vs. non-hypoglycaemia). In contrast, the operating threshold refers to the threshold selected for practical use based on clinically meaningful trade-offs between hypoglycaemia detection (sensitivity) and false-alert burden.

Decision thresholds were determined using a validation-driven, deployment-oriented strategy. Given the severe class imbalance (approximately 4% event prevalence) and the clinical asymmetry between missed hypoglycaemic events and false alarms, operating thresholds were selected within a sensitivity-oriented regime. Specifically, thresholds were optimised on the validation set using maximisation of the F1 score in precision–recall space (PR–F1), with Youden’s *J* statistic reported as a secondary reference.

Calibration was assessed using the Brier score and expected calibration error (ECE), complemented by reliability diagrams comparing predicted and observed event rates [31,32].

Statistical uncertainty was quantified using participant-level bootstrap resampling (2000 replicates) to derive 95% confidence intervals for AUC and F1, accounting for within-participant correlation induced by overlapping sequences. As a sensitivity analysis, pairwise differences in ROC AUC between models were compared using the DeLong method for correlated ROC curves [33].

#### 2.7.3. Robustness, Temporal Stability, and Interpretability

Cross-phase robustness was examined by training models on Ramadan data and testing on the post-fasting month, and vice versa, within the held-out participant splits, with PR AUC and recall averaged across directions to characterise performance under covariate shift [34]. A balanced (1:1) diagnostic subset was analysed to inspect error symmetry independently of prevalence effects.

Temporal alert stability was evaluated across horizons from 1 to 12 h using an N-of-M rule requiring at least two positive predictions within three consecutive windows, reducing sensitivity to isolated probability spikes [35].

Model explainability was assessed using SHAP values computed with DeepExplainer, with GradientExplainer used as a fallback when required. Global SHAP summary plots identified the most influential features across the cohort, while per-patient SHAP profiles illustrated personalised temporal risk patterns linking hypoglycaemia risk to CGM variability, activity, and sleep features (Appendix C).

#### 2.7.4. Reproducibility

All analyses were implemented in Python 3.11 using fixed random seeds to ensure reproducibility. Model training, preprocessing, and evaluation scripts are version-controlled and available in a public repository, with exact package versions documented in the accompanying materials.

#### 2.7.5. Validation

Despite the use of patient-wise, leak-free evaluation, no prospective or external validation was conducted. Accordingly, the current evaluation is limited to retrospective observational data and should be interpreted within this context.

To maintain clarity in the main manuscript while preserving methodological transparency, detailed analyses (including balanced test set evaluation and extended validation results) are provided in the Appendix D and Appendix E.

## 3. Results

### 3.1. Cohort Characteristics, Glycaemic Patterns, and Feature Behaviour

**Figure A1:**
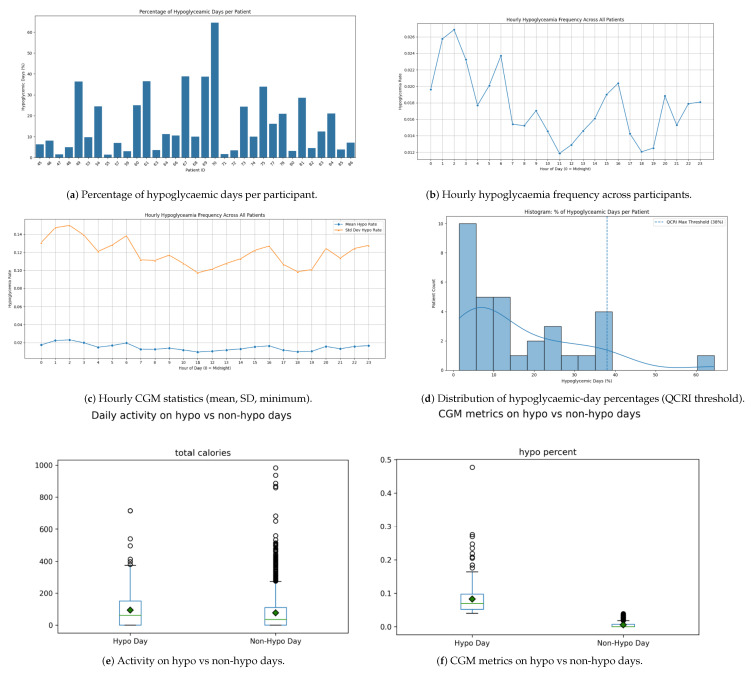

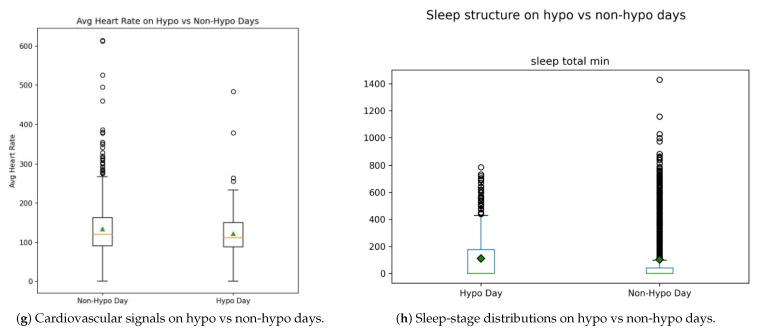
Participant-level and temporal patterns of hypoglycaemia during Ramadan. Panels (**a**,**b**) show inter-individual variability and circadian hypoglycaemia patterns. Panels (**c**,**d**) summarise hourly CGM dynamics and hypoglycaemia burden. Panels (**e**–**h**) compare behavioural, glycaemic, cardiovascular, and sleep-related features between hypoglycaemic and non-hypoglycaemic days.

Thirty-three adults with type 1 diabetes contributed analysable CGM and wearable data spanning Ramadan and the subsequent month. Hypoglycaemia burden showed substantial between-participant heterogeneity: the distribution of hypoglycaemic days was positively skewed, with a median of 8% (range 1.4–67.7%), and two individuals exceeded the QCRI reference threshold of 38% hypoglycaemic days. This variability highlights that fasting-related physiology and behaviour can manifest differently across individuals, even within a relatively homogeneous clinical cohort. Descriptive summaries were generated to characterise hypoglycaemia burden and its temporal distribution prior to modelling. Figure A1a,b show participant-level variability in hypoglycaemic days and the hourly distribution of hypoglycaemic events across the cohort.

The thresholds reported in this section correspond to operating thresholds, i.e., deployment-oriented decision points selected to balance hypoglycaemia detection and alert burden, rather than arbitrary classification cut-offs. Sensitivity analyses demonstrated that model performance and class-specific metrics remained stable across a range of operating thresholds (0.40–0.60), supporting the robustness of the selected decision boundary.

At the cohort level, CGM traces exhibited a clear circadian structure, with early-morning glucose nadirs during prolonged fasting and higher evening variability after iftar (Figure A1c,e). Hypoglycaemia burden also varied markedly across participants, with most individuals experiencing low percentages of hypoglycaemic days and a small subgroup exceeding the QCRI threshold (Figure A1d). Comparisons between hypoglycaemic and non-hypoglycaemic days indicated that differences extended beyond glucose alone: activity levels were typically lower, CGM variability was higher, and modest but consistent shifts were observed in heart rate, oxygen saturation, and sleep structure (Figure A1f). Together, these multimodal patterns suggest that hypoglycaemia during Ramadan occurs within a broader behavioural and physiological context rather than being driven by isolated glucose fluctuations.

**Figure A2:**
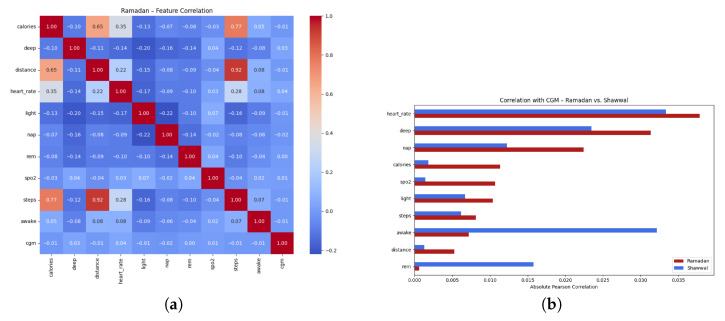
Diagnostic overview of multimodal feature behaviour. Panel (**a**) shows clustering patterns among lifestyle and physiological signals during Ramadan, supporting PCA-driven dimensionality reduction. Feature–feature correlation heatmap for the Ramadan period. Activity variables cluster strongly, consistent with the PCA structure (Table A6). Panel (**b**) compares Random Forest feature-importance rankings across Ramadan and the after-fasting month, highlighting the dominant contribution of activity and cardiorespiratory markers to glycaemic variation. Random Forest feature-importance ranking for Ramadan (red) and the after-fasting month (blue). Heart rate, calories, and steps remain the most influential wearable predictors of CGM dynamics.

**Figure A3:**
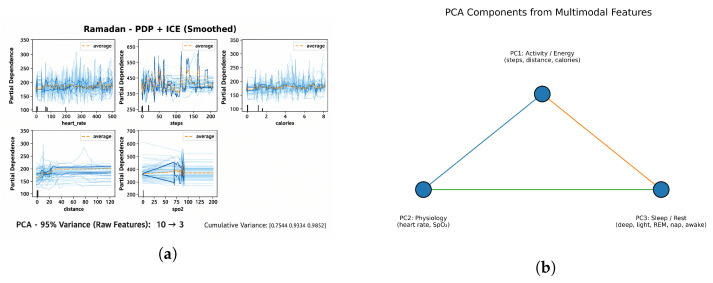
Feature interpretability analyses. Panel (**a**) presents representative partial dependence and individual conditional expectation (ICE) profiles summarizing cumulative PCA variance during Ramadan. These plots illustrate the influence of individual features on model predictions at both the population and instance levels. Panel (**b**) provides an overview of the three principal components underlying the multimodal dataset, offering a conceptual representation of PCA-derived structure from CGM and wearable features. Specifically, PC1 (activity/energy), PC2 (physiology), and PC3 (sleep/rest) capture the dominant sources of variability across fasting and post-fasting transitions.

As detailed in the Methods, the final dataset comprised 1164 participant-days with approximately 4% hypoglycaemic hours, corresponding to a pronounced 1:23 class imbalance. As a sensitivity analysis, we evaluated alternative lookback window lengths (6, 12, 18, 24, 36, 72, and 84 h). Model performance improved progressively up to a 36 h window, after which discrimination and calibration metrics (ROC AUC, PR AUC, and recall) stabilised. Longer windows (72 and 84 h) yielded similar but not superior performance. These findings support the 36 h lookback window as an efficient configuration that captures relevant temporal dynamics without introducing unnecessary model complexity. The effect of temporal context is further illustrated in Figure A8d, where performance improves with increasing lookback window up to 36 h before stabilising. Feature diagnostics indicated that the input space was relatively clean and *non-redundant*, with variance inflation factors generally close to 1. As an exploratory benchmark, Random Forest models consistently ranked heart rate, calories, and step count among the most informative wearable signals related to glycaemic variation, while sleep-stage features contributed more modestly (Table A6). Principal component analysis further clarified these relationships, confirming that wearable signals clustered into activity/energy, cardiorespiratory physiology, and sleep/rest domains, and that CGM-derived statistics condensed into components dominated by glucose amplitude and variability (Figure A2 and Figure A3). These findings motivated the use of compact multimodal representations in the sequence models.

Sensitivity analyses examining the impact of dimensionality reduction showed that removing the third PCA component from CGM and wearable feature blocks did not materially affect model performance. Discrimination (ROC AUC, PR AUC), calibration (Brier score), and recall remained stable across configurations, indicating that predictive performance was not dependent on low-variance components.

**Figure A4:**
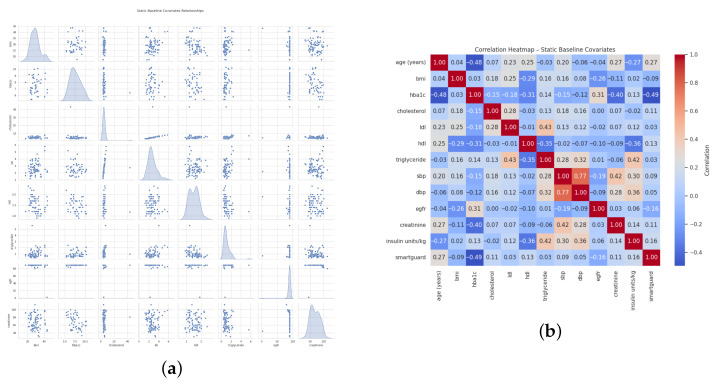
Static baseline covariate relationships. Panel (**a**) shows pairwise scatter and density distributions; panel (**b**) displays the corresponding correlation matrix, highlighting coherent cardiometabolic and renal associations across participants.

**Figure A5:**
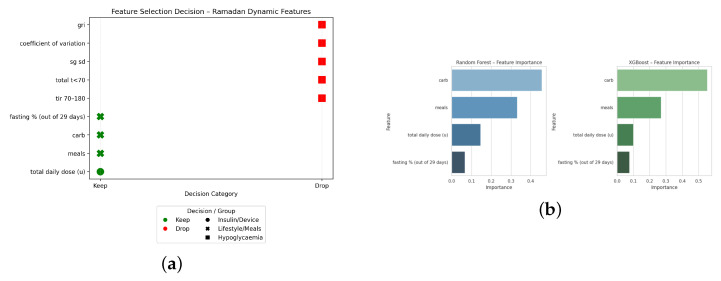
Feature dependencies and predictive relevance of Ramadan-specific dynamic variables. Panel (**a**) shows correlations between insulin delivery, device metrics, and lifestyle-related variables. Panel (**b**) presents ensemble-derived feature importance scores, highlighting bolus%, auto-basal%, total daily dose, meals/day, carbohydrate intake, and fasting% as key predictors of hypoglycaemia exposure (total time below 70 mg/dL).

Static baseline characteristics depicted a young cohort with generally preserved metabolic and renal health and high SmartGuard engagement throughout the study (Table A3; Figure A4). Ramadan-phase visit-level variables captured meaningful behavioural and therapeutic adjustments, with bolus%, auto-basal%, total daily dose, carbohydrate intake, meals per day, and fasting% showing the strongest associations with hypoglycaemia burden (Figure A5). Collectively, these observations reinforce that both short-term behaviour and longer-term clinical characteristics shape the risk landscape in which hypoglycaemia develops during Ramadan.

Across all experiments, the strongest-performing models were consistently obtained under the non-resampled setting, indicating that the combination of CGM-derived features, temporal encodings, and Ramadan-specific behavioural proxies provided sufficient discriminatory signal without requiring synthetic class balancing. These findings suggest that preserving the original temporal structure and event distribution is more beneficial than applying resampling techniques in this context.

The final Ramadan dataset comprised 19,687 hourly observations with 65 engineered features. Using a 36 h sequence window, a total of 12,870 training, 1991 validation, and 3638 test sequences were generated. The dataset exhibited pronounced class imbalance, with approximately 4% hypoglycaemic events.

### 3.2. Model Performance

#### 3.2.1. Primary Model Performance on the Original Diagnostic Test Set

On the original, naturally imbalanced diagnostic test set, the full multimodal LSTM model with 50 units achieved strong discriminative performance. At the selected operating threshold, the model attained an ROC AUC of 0.87 and a PR AUC of 0.34, with sensitivity for next-hour hypoglycaemia of approximately 0.77 and a weighted F1-score of 0.88 (Table 3). These results indicate effective minority-class detection under clinically realistic class imbalance.

**Figure 2:**
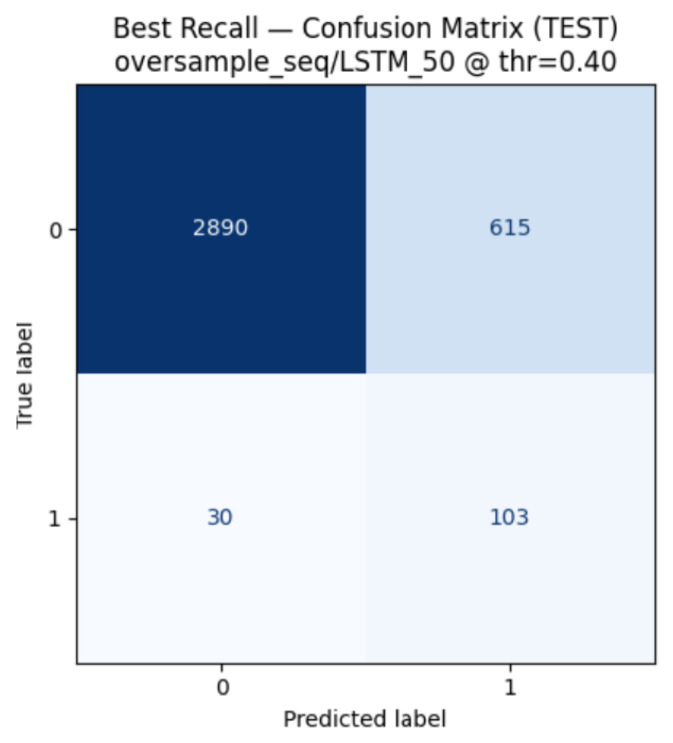
Confusion matrix for the full multimodal model (all features) under the original test distribution.

Evaluation under a balanced diagnostic test set yielded comparable performance, with ROC AUC remaining stable (0.86) and sensitivity preserved, alongside improved precision and overall accuracy (0.79). The corresponding confusion matrix for the full multimodal model is shown in Figure 2, illustrating balanced error characteristics under equal class prevalence. At this sensitivity-oriented operating point, the model generated 718 positive alerts over 3638 evaluated hours, corresponding to approximately 4.7 alerts per patient-day (about 4.1 false alerts and 0.7 true alerts per day).

Models incorporating wearable-derived behavioural and physiological signals provided additional predictive value beyond CGM alone. In particular, BiLSTM configurations integrating wearable features achieved ROC AUC values of approximately 0.92 and PR AUC up to 0.52 on the original test set, exceeding the precision–recall performance of CGM-only models (PR AUC 0.31–0.35) despite similar ROC AUC values (Table 3). These findings indicate that wearable-derived behavioural and physiological signals contribute substantially to near-term hypoglycaemia risk prediction.

As shown in Table 3, the Temporal + Static configurations achieved the strongest overall performance on the original, naturally imbalanced diagnostic test set, outperforming other feature groups across discrimination and calibration metrics. In particular, the no-resampling Temporal + Static LSTM 100 model at a threshold of 0.40 achieved the best overall results, with an accuracy of 0.966, an F1-score of 0.468, a precision of 0.539, a recall of 0.414, a specificity of 0.987, an ROC AUC of 0.909, a PR AUC of 0.454, and the lowest Brier score (0.025). These findings indicate that temporally enriched multimodal modelling improved both discrimination and probability calibration under clinically realistic class imbalance.

Within the Temporal + Static group, the LSTM 100 configuration consistently outperformed the LSTM 50 and BiLSTM variants, yielding the highest ROC AUC, PR AUC, and precision, while maintaining comparable recall and specificity. Although CGM + wearable models achieved higher recall in some configurations, their precision and calibration were generally weaker, resulting in a less balanced overall performance profile.

Consistent with the temporal sensitivity analysis, models incorporating extended temporal context (36 h lookback) demonstrated superior performance compared to shorter-context or non-temporal configurations. This improvement reflects the ability of temporally enriched models to capture delayed physiological and behavioural effects, contributing to improved performance under severe class imbalance.

A comprehensive comparison across resampling strategies and feature configurations on the original diagnostic test set is reported in Table A10, with full ROC and PR curves provided in Figure A7a–d. As illustrated in Figure A8a,b, multimodal and CGM + wearable configurations consistently outperform CGM-only models in both ROC AUC and precision–recall space.

Notably, PR AUC values varied more substantially across feature configurations than ROC AUC, underscoring the importance of precision–recall analysis under severe class imbalance.

#### 3.2.2. Calibration

Calibration quality differed noticeably across model configurations on the imbalanced test set. Across the evaluated configurations in Table 3, Brier scores ranged from 0.049 to 0.100. The static+wearable LSTM 50 was the most well-calibrated model (Brier = 0.049), followed by the LSTM 100 family (Brier ≈ 0.058). The multimodal BiLSTM configurations showed moderate probability calibration (Brier ≈ 0.065), whereas CGM-only models exhibited the largest calibration errors (Brier ≈ 0.077–0.095), indicating reduced probability stability when relying on CGM inputs alone.

**Figure 3:**
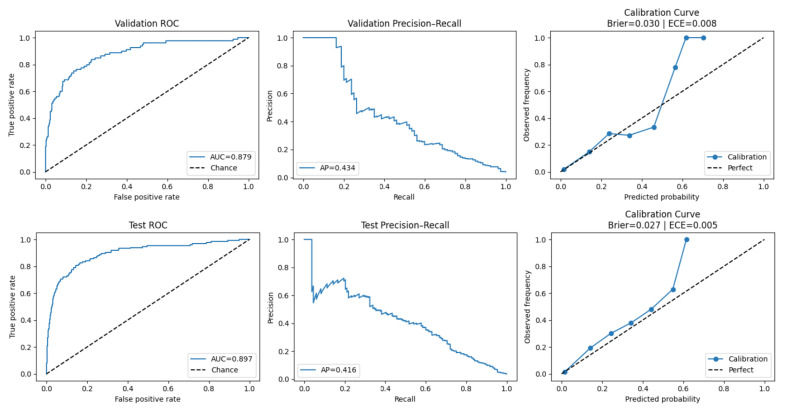
Validation (**top row**) and test (**bottom row**) performance of the leak-safe multimodal BiLSTM. Columns show ROC curves (**left**), precision recall curves (**middle**), and reliability diagrams (**right**). Area-under-curve values (AUC and average precision, AP) are indicated in the legends. Brier scores and expected calibration error (ECE) demonstrate good calibration on both validation (Brier = 0.030, ECE = 0.008) and test data (Brier = 0.027, ECE = 0.005).

For a representative deployment-relevant configuration, we further examined reliability diagrams and expected calibration error (ECE) for the selected leak-safe multimodal BiLSTM (Figure 3). This model achieved Brier = 0.030 (ECE = 0.008) on the validation set and Brier = 0.027 (ECE = 0.005) on the held-out test set, indicating close alignment between predicted probabilities and observed event frequencies under natural prevalence. On the balanced diagnostic subset (1:1 prevalence; diagnostic only), Brier scores increased to approximately 0.15–0.17 (Table A9), while discrimination remained similar.

#### 3.2.3. Model Comparison Under Balanced Training and Testing

Using the leak-free, patient-wise pipeline described in Appendix D, we compared model architectures, feature sets, and resampling strategies under both natural prevalence and a balanced diagnostic setting (Table 3; Table A9 and Table A10). On the original held-out test set, the multimodal LSTM 50 trained with sequence-level oversampling (all features: static + visit-level + wearable + CGM) provided the most favourable compromise between discrimination and sensitivity for an early-warning application. At the sensitivity-oriented operating point τ=0.40, this configuration achieved ROC AUC = 0.867 and PR AUC = 0.340, detecting approximately 77% of hypoglycaemic hours (Accuracy = 0.77) with Precision = 0.14 under severe class imbalance.

Across feature subsets, multimodal and wearable-enhanced models consistently outperformed CGM-only configurations. CGM + Static + wearable models achieved high overall accuracy and minority-class F1 values up to approximately 0.35, but at the cost of lower recall (0.46–0.53). CGM + wearable models retained strong discrimination, with top-performing configurations (including BiLSTM and LSTM 100) achieving ROC AUC values in the range of approximately 0.90–0.915 and PR AUC up to approximately 0.52 (0.522), indicating that activity and cardiorespiratory physiology alone provide substantial predictive signal. By contrast, CGM-only models showed comparable ROC AUC but consistently lower PR AUC, highlighting that ROC AUC alone can overstate performance in rare-event settings.

Evaluation on the balanced 1:1 diagnostic subset (diagnostic only) showed substantially higher minority-class performance across all architectures, with F1 values rising to approximately 0.78–0.86 and PR AUC exceeding 0.86 (Table A9). These results indicate that event scarcity, rather than model capacity, is the primary limitation under real-world prevalence.

Resampling strategy influenced the recall–precision trade-off in a predictable manner. Sequence-level oversampling consistently improved recall and PR AUC in multimodal and CGM + wearable models, aligning with the clinical priority of minimising missed hypoglycaemic events. In contrast, SMOTE and SMOTE + ENN variants yielded more conservative operating points with higher precision and accuracy but reduced recall. Undersampling improved sensitivity in some cases but could reduce ROC AUC by discarding informative majority-class data, consistent with participant-level bootstrap AUC comparisons (Table A10).

**Impact of sampling strategies in the extended temporal-feature experiment.** The impact of different sampling strategies revealed distinct trade-offs between precision and recall. Models trained without resampling consistently achieved the best balance, indicating robust performance under the natural class distribution.

Oversampling strategies increased recall (up to approximately 0.56) but at the cost of reduced precision (approximately 0.30), leading to a higher rate of false positives. In contrast, undersampling achieved higher precision (up to approximately 0.80) but substantially reduced recall, particularly in the temporal-feature setting.

Although undersampling produced higher F1-scores (up to approximately 0.77) under artificially balanced conditions, this improvement reflects distribution distortion rather than true generalisation. In comparison, non-resampled models demonstrated more stable performance across validation and test sets, indicating superior robustness.

Overall, the extended temporal-feature experiment demonstrates that incorporating temporal dynamics and Ramadan-specific behavioural proxies significantly improves hypoglycaemia prediction without requiring aggressive class rebalancing. The strong performance of models trained on the original imbalanced dataset suggests that temporal structure provides sufficient discriminatory information to mitigate class imbalance effects.

The discrepancy between the selected model (BiLSTM) and the highest-performing test model (LSTM 100) underscores the importance of strict validation-based model selection to ensure unbiased evaluation.

Finally, the substantial performance gains observed at the aggregated level emphasise the importance of temporal context in modelling hypoglycaemia risk and support the use of clinically interpretable risk summaries for deployment.

#### 3.2.4. Impact of Resampling on Hypoglycaemia Discrimination

Overall, bootstrap analyses indicated that performance differences were modest in absolute magnitude but statistically significant in selected pairwise contrasts. In particular, models trained without resampling using an LSTM with 100 units achieved higher ROC AUC than an undersample_seq/BiLSTM configuration (ΔAUC = 0.037, p<0.001), indicating a measurable degradation in discrimination under aggressive undersampling (Table A11).

On the imbalanced test set, the model achieved ROC AUC = 0.867, PR AUC = 0.341, and Recall_1_ = 0.77, based on participant-level bootstrap resampling (2000 replicates).

By contrast, sequence-level oversampling yielded intermediate AUC values that were modestly but significantly higher than undersampling-based approaches (ΔAUC = 0.024, p=0.037), while remaining statistically comparable to the no-resampling baseline. These findings support sequence-level oversampling as a pragmatic compromise when the clinical priority is to minimise missed hypoglycaemic events without incurring substantial losses in overall discrimination. Although the absolute AUC differences were small, their consistency across bootstrap resamples and alignment with recall-oriented metrics indicates practical relevance in safety-critical settings.

#### 3.2.5. Threshold Behaviour

Decision thresholds selected during validation were consistently concentrated within a narrow range (0.40–0.60) across model architectures and feature configurations, indicating limited sensitivity to threshold choice. When these fixed thresholds were applied to both the original and balanced diagnostic test sets, discriminative performance and class-specific metrics remained largely stable. This corresponds to approximately 23% of hypoglycaemic events not being detected at the selected operating point. This behaviour reflects the expected trade-off between sensitivity and false-alert burden under severe class imbalance, where improving event detection necessarily increases the likelihood of false positives.

In the balanced diagnostic test set, the precision for the hypoglycaemia class increased in all configurations, while recall was preserved at levels comparable to those observed under the original class distribution. This trade-off yielded systematically higher F1 scores without evidence of degradation in recall. Balanced-set Brier scores ranged from 0.13 to 0.21, indicating maintained probabilistic calibration under class reweighting.

Overall, threshold stability across distributions supports the robustness of the learned decision boundaries and suggests that threshold tuning artefacts do not drive performance gains under balancing. Full threshold-sensitivity results for the balanced diagnostic test set are reported in Table A9. The trade-off between recall and alert burden across thresholds is shown in Figure A8c, highlighting the expected increase in false alerts at sensitivity-oriented operating points.

### 3.3. External Cross-Phase Validation

To assess external generalisation across physiological states, we evaluated cross-phase performance by training models on fasting-period data (Ramadan) and testing on the post-fasting period (Shawwal), and vice versa. Although conducted within the same cohort, this analysis probes robustness under a meaningful shift in behaviour, sleep, and metabolic context associated with fasting. Cross-phase transfer preserved both discrimination and calibration, indicating robustness under fasting-to-post-fasting behavioural shift.

Across architectures, recurrent models retained strong discriminative performance under cross-phase transfer. The BiLSTM achieved the highest and most consistent performance, with ROC AUC values of 0.917 for Ramadan → Shawwal and 0.911 for Shawwal → Ramadan, together with corresponding PR AUC values of 0.501 and 0.481 and low Brier scores (approximately 0.03). These results indicate that predictive performance and probability calibration were largely preserved when models were applied across fasting and post-fasting conditions.

Complete cross-phase validation results for all recurrent architectures and both transfer directions are reported in Table A12.

### 3.4. Explainability and Patient-Level Behaviour

To support interpretability of the proposed framework, we combined selective SHAP-based analyses with patient-level visualisations illustrating how predicted hypoglycaemia risk evolves over time. Because full-sequence SHAP computation is computationally expensive for recurrent architectures, explainability analyses were conducted selectively for representative models and patients, complemented by longitudinal risk trajectories at the individual level.

**Figure 4:**
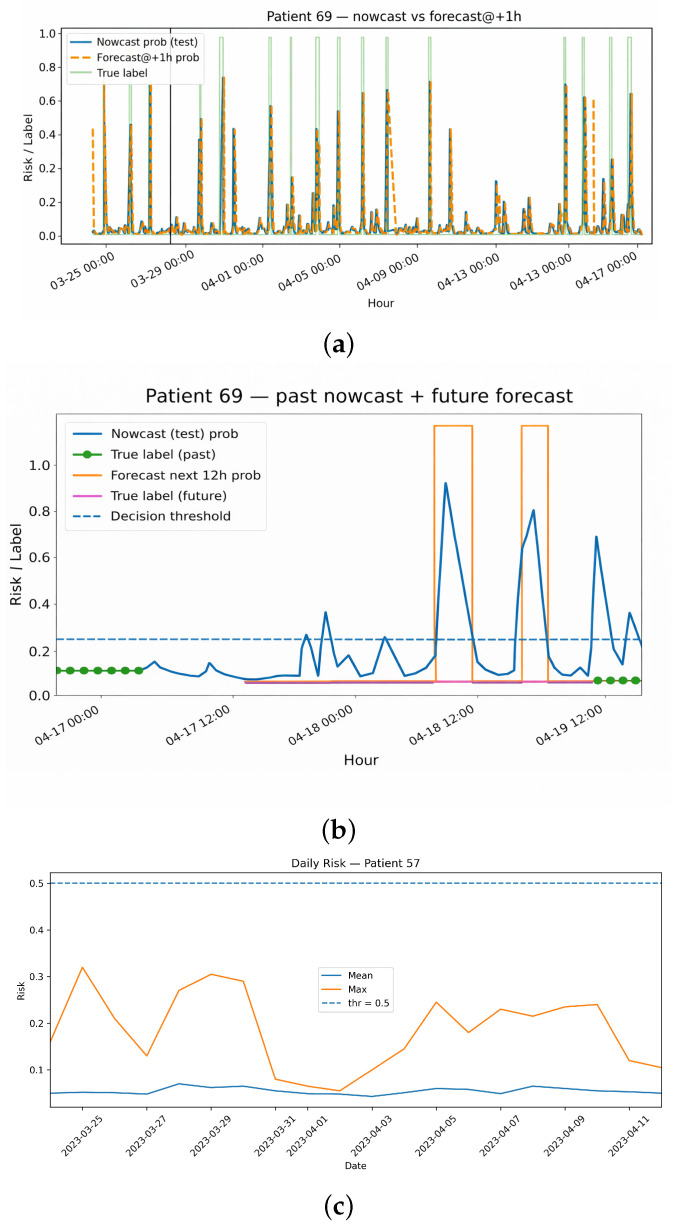
Combined visualisation of risk estimates across nowcast, forecast, and daily summaries for selected patients. Patient-level risk trajectories and 1-h-ahead prediction. (**a**) **Patient 69: nowcast vs. +1 h forecast** The blue curve represents the *nowcast*, defined as the model’s estimated probability that the current hour contains at least one CGM value ≤70 mg/dL, based on the preceding 36 h of CGM and wearable data. The orange curve represents the *+1 h forecast*, i.e., the predicted probability that the next hour will contain any CGM value ≤70 mg/dL, corresponding to a clinically actionable one-hour prediction horizon. The horizontal dashed line denotes the decision threshold (τ), above which alerts are triggered. Vertical bars indicate hours in the held-out test set where hypoglycaemia was observed. (**b**) **Patient 69: past nowcast + 12 h forecast.** The left segment shows historical nowcast probabilities over the preceding 36 h, while the right segment shows a rolling 12 h forecast horizon constructed from consecutive +1 h predictions. The dashed horizontal line denotes the decision threshold (τ). Orange shaded regions indicate predicted high-risk windows where forecast probabilities exceed τ, corresponding to potential alerts. Green markers represent the observed true labels across the timeline. This visualization demonstrates how model outputs can support real-time risk monitoring and prospective alerting. Vertical separation between past and forecast segments reflects the transition from observed data to prospective prediction. (**c**) **Patient 57: daily hypoglycaemia risk** Daily risk is summarised using the mean (blue) and maximum (orange) predicted probabilities per day, together with the number of evaluated windows and observed hypoglycaemic hours. The dashed line indicates the decision threshold (). Days with observed hypoglycaemia exhibit higher mean and peak predicted risk, demonstrating alignment between model predictions and clinically relevant patterns of risk.

At each hour *t*, the model produces two related probability estimates. The *nowcast* represents the estimated probability that the *current* hour *t* contains at least one CGM value ≤ 70 mg/dL, while the *forecast* represents the estimated probability that the *next* hour t+1 will contain any CGM value ≤ 70 mg/dL. Both estimates are computed using only the preceding 36 h of CGM and wearable data, such that the +1 h forecast provides a genuine 60-minute early-warning window during which insulin dosing, carbohydrate intake, or activity may be adjusted. In Figure 4a,b, the blue curve represents “current-hour risk”, while the orange curve represents “next-hour risk given no intervention”. Hereafter, τ denotes the decision threshold selected on the validation set.

Figure 4a illustrates **hour-by-hour nowcast probabilities for Patient 69** together with the calibrated decision threshold, with vertical markers indicating hours labelled as hypoglycaemic in the held-out test set. The corresponding +1 h forecast follows a similar temporal pattern but is shifted forward by one hour, highlighting the advance warning provided by the model. Figure 4b extends this view by combining historical nowcasts with a rolling 12 h forecast sequence, illustrating how near-term risk is projected over a half-day horizon from a given reference point.

**Daily risk summaries were examined using aggregated profiles for Patient 57** (Figure 4c). For each day, we computed the number of evaluated windows, the number of true hypoglycaemic windows, the number of predicted positive windows, and the mean and maximum predicted risk (Table A13). Days with non-zero hypoglycaemia prevalence consistently exhibited higher mean and peak predicted risk than days without observed events. Similar patterns were observed across additional individuals (e.g., patients 67, 69, 80, and 85), indicating that patient-level risk trajectories align with clinically recognisable patterns of hypoglycaemia burden.

**Figure A6:**
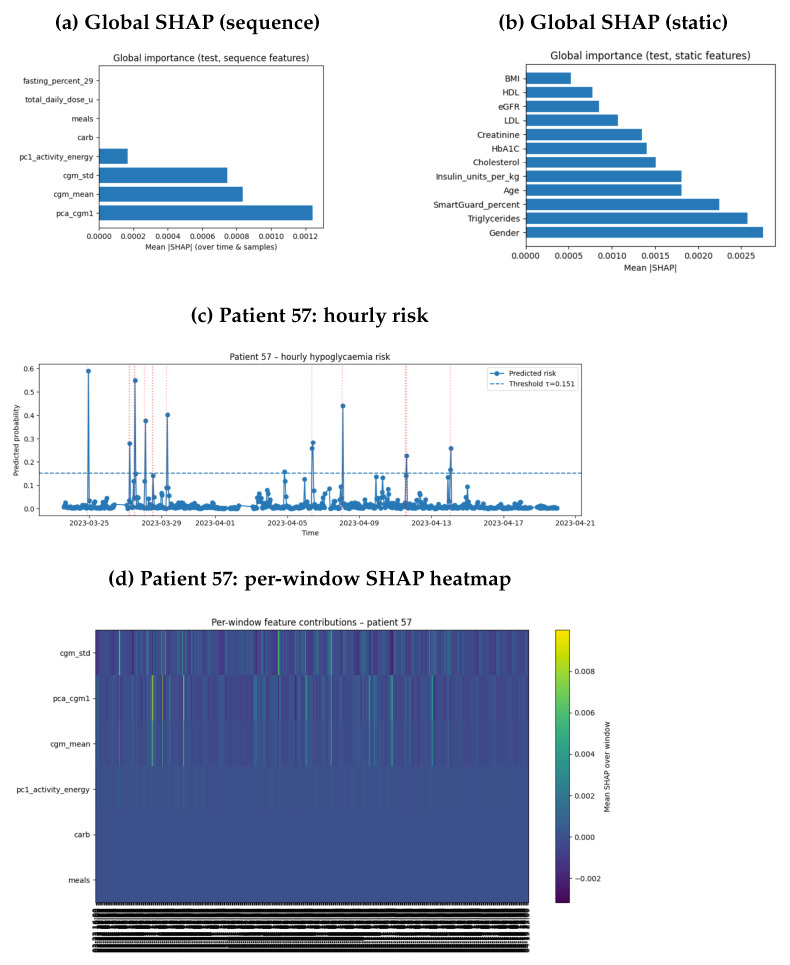
Explainability diagnostics for the multimodal BiLSTM. (**a**) Global SHAP importance for sequence features on the test set (mean |SHAP| aggregated over time and samples). (**b**) Global SHAP importance for static baseline features on the test set. (**c**) Hourly hypoglycaemia risk for patient 57; the dashed line denotes the calibrated decision threshold (τ=0.151), and vertical markers indicate hours labelled as hypoglycaemic. Red vertical dashed lines denote the occurrence of true hypoglycaemia events, defined using CGM-derived glucose values below the clinical threshold. These annotations allow visual assessment of the temporal alignment between predicted risk peaks and actual hypoglycaemia episodes. (**d**) Per-window SHAP contributions (mean SHAP over each 36 h window) for patient 57, showing that risk spikes align with higher contributions from CGM amplitude and variability features.

**Figure A7:**
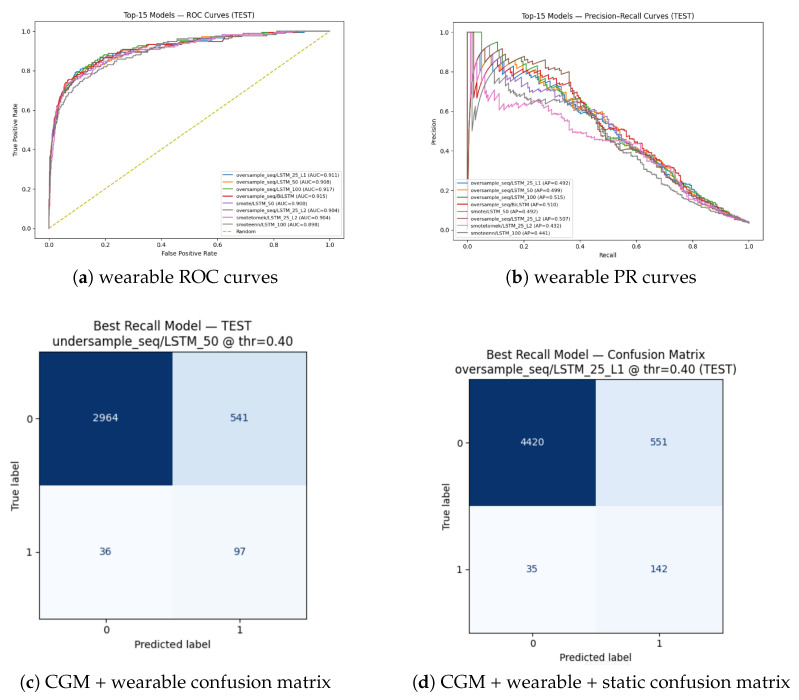
CGM + wearable-model performance diagnostics under the original held-out test distribution. (**a**) ROC curves for the top-performing CGM + wearable configurations ranked by weighted F_1_. (**b**) Precision–recall curves for the same configurations. (**c**) Confusion matrix for the CGM + wearable model. (**d**) Confusion matrix for the CGM + wearable + static model.

**Figure A8:**
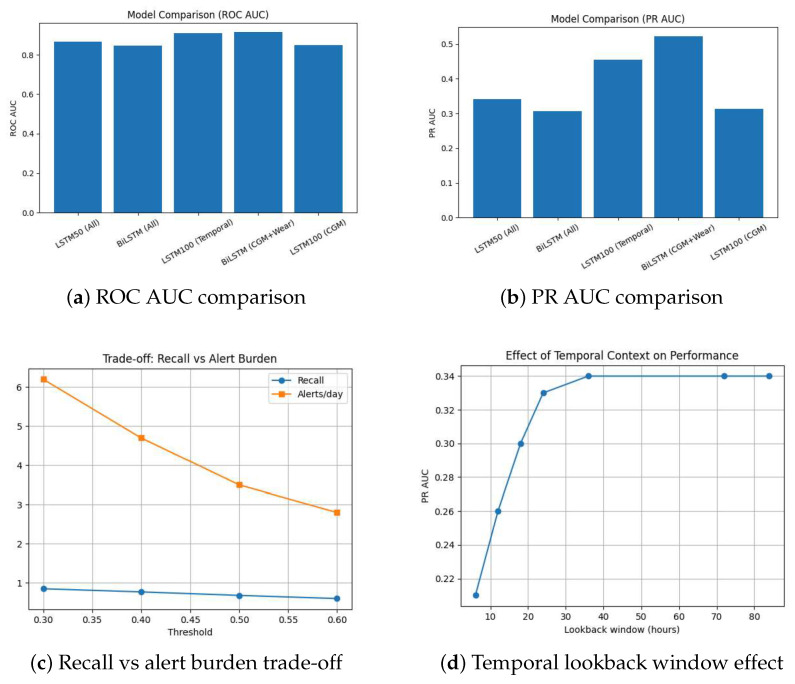
**Supplementary model diagnostics and performance analyses.** (**a**) ROC AUC comparison across representative models. (**b**) Precision–recall AUC (PR AUC) comparison highlighting improved minority-class performance with multimodal and CGM + wearable configurations. (**c**) Trade-off between recall and alert burden across decision thresholds, illustrating the clinical balance between hypoglycaemia detection and false alerts. (**d**) Effect of temporal lookback window on performance, showing improvement up to 36 h followed by saturation.

**Selective SHAP analyses** were used to provide qualitative insight into feature contributions for representative models and sequences. Global SHAP importance for the multimodal BiLSTM is summarised in Figure A6a,b, while patient-level risk trajectories and per-window SHAP attributions are shown in Figure A6c,d. These analyses indicate that short-term CGM amplitude and variability dominate sequence-level risk estimation, while baseline cardiometabolic and device-use features modulate personalised risk profiles.

**Daily aggregated risk prediction in the extended temporal-feature experiment.** At the daily aggregation level, prediction performance improved substantially. Across 165 patient-days, the selected model achieved an F1-score of 0.724, with precision of 0.792 and recall of 0.667. This improvement indicates that hypoglycaemia risk is more effectively captured as a temporal process rather than as isolated hourly events. Temporal aggregation enhances predictive stability and clinical interpretability, supporting its relevance for real-world decision-making during Ramadan fasting.

## 4. Discussion

### 4.1. Principal Findings

This study demonstrates that behaviour-aware, multimodal recurrent models can deliver calibrated, hour-ahead hypoglycaemia risk estimates in free-living adults with type 1 diabetes fasting during Ramadan. By integrating continuous glucose monitoring (CGM) with wrist-worn wearable signals, visit-level behavioural variables, and static clinical characteristics, we move beyond CGM-centric prediction toward a richer representation of fasting-related physiology and behaviour.

Model performance was robust to dimensionality reduction choices, indicating that predictive signals were primarily captured by dominant principal components. The selection of the 36 h lookback window reflects a balance between physiological relevance and computational efficiency, supported by empirical sensitivity analysis demonstrating performance saturation beyond this range.

The improved performance of temporally enriched models highlights the importance of capturing extended behavioural and circadian patterns for hypoglycaemia prediction, particularly in the context of Ramadan fasting, where metabolic dynamics extend beyond short-term time horizons.

The models were trained and evaluated using patient-wise, leak-free splits and class-weighted focal loss under the natural data distribution, in which hypoglycaemia comprised approximately 4% of hourly observations (≈1:23 imbalance). We prioritised evaluation metrics appropriate for rare outcomes—precision–recall AUC (PR AUC), hypoglycaemia-class recall, and probability calibration—alongside ROC AUC.

Under these demanding conditions, a compact multimodal LSTM 50 achieved an ROC AUC of 0.867 and PR AUC of 0.34 on the imbalanced test set, identifying approximately 77% of next-hour hypoglycaemic events at a clinically meaningful operating threshold. These results demonstrate that actionable, hour-ahead hypoglycaemia prediction is feasible even under severe class imbalance and fasting-related behavioural disruption.

#### 4.1.1. Added Value of Multimodal and CGM + Wearable Models

Across feature configurations, multimodal architectures consistently outperformed CGM-only models. Incorporating wearable-derived activity, cardiovascular physiology, sleep context, and visit-level behavioural variables improved discrimination, calibration, and sensitivity to imminent hypoglycaemia compared with relying on glucose trajectories alone. Notably, recurrent models incorporating wearable-derived features—particularly BiLSTM configurations—achieved the highest discrimination on the original test distribution (ROC AUC 0.915; PR AUC 0.522), highlighting the predictive value of behavioural and physiological context. Nonetheless, precision and minority-class F1 scores remained modest under natural prevalence, despite high AUC values, reflecting the inherent constraints on positive predictive value when hypoglycaemic hours are rare. These findings indicate that multimodal context adds genuine predictive signal beyond CGM, while underscoring that limited precision is a structural consequence of event rarity rather than model failure. This behaviour is physiologically plausible, as wearable signals capture autonomic, activity-related, and sleep-associated dynamics that may precede glucose declines and generalise across fasting and non-fasting states.

#### 4.1.2. Rare-Event Metrics and the Role of Balanced Evaluation

Performance on the naturally imbalanced test set could appear modest if interpreted using precision-based metrics alone. When the same models were evaluated on a 1:1 balanced diagnostic subset, PR AUC and hypoglycaemia-class F1 increased markedly across all architectures, while recall remained stable. For example, the all-features LSTM 50 showed a marked improvement in precision–recall performance and minority-class F1 when evaluated under balanced conditions, indicating that the learned decision boundaries were robust and not driven by threshold artefacts. Similar patterns were observed for CGM-only and BiLSTM models.

Under an event prevalence of approximately 4%, achieving high recall necessarily entails a substantial number of false positives; this behaviour reflects a fundamental prevalence constraint rather than a modelling deficiency. These results indicate that low apparent precision under natural prevalence was driven primarily by event scarcity rather than poor discrimination, and they illustrate why ROC AUC alone can overstate performance in rare-event prediction. Accordingly, PR-based metrics and calibration are essential for realistic appraisal of hypoglycaemia risk models.

#### 4.1.3. Cross-Phase Generalisability and Physiological Plausibility

Training phase influenced generalisation across fasting states. Models trained on Ramadan data transferred more effectively to the post-fasting month than the reverse, with the multimodal BiLSTM achieving ROC AUC 0.917 and PR AUC 0.501 under Ramadan → post-fasting evaluation, alongside good calibration (Brier ≈ 0.029). This asymmetry is physiologically plausible: fasting days exhibit more structured circadian patterns—early-morning glucose nadirs and post-iftar recovery—that provide clearer, repeatable signals for learning than the more variable physiology observed after Ramadan. Patient-level risk trajectories reinforce this interpretation, with predicted risk typically rising well in advance of observed hypoglycaemic episodes and remaining low on event-free days.

#### 4.1.4. Effects of Imbalance-Handling Strategies on Sensitivity and Alert Burden

Imbalance-handling strategies shaped the trade-off between sensitivity and alert burden in predictable ways. Sequence-level oversampling consistently improved recall and PR AUC in multimodal and CGM + wearable models, producing behaviour well suited to early-warning or screening applications where missed hypoglycaemic events are clinically unacceptable. In contrast, SMOTE-based approaches tended to increase precision and overall accuracy at the cost of reduced recall, yielding more conservative models with fewer alerts but greater risk of missed events. Undersampling often degraded ROC AUC by discarding informative non-hypoglycaemic data. Overall, these patterns suggest that simple oversampling offers a pragmatic compromise when prioritising safety in rare-event detection.

#### 4.1.5. Methodological Choices Underpinning Reliability

Several design choices were central to the robustness of these findings. Patient-wise splits and leak-free preprocessing ensured genuine generalisation to unseen individuals. Multimodal feature integration enabled the models to capture both short-term glycaemic dynamics and longer-term behavioural and clinical context—an approach that remains uncommon in Ramadan-focused machine-learning studies. Class-weighted focal loss stabilised optimisation under severe class imbalance without excessive reliance on resampling. Balanced diagnostic evaluation and explicit cross-phase testing further disentangled intrinsic model capability from prevalence effects and behavioural shift. Finally, PCA-based dimensionality reduction and SHAP analyses indicated that predictions were grounded in physiologically coherent components of glucose amplitude and variability, supported by activity and selected baseline characteristics.

From a clinical perspective, approximately 23% of hypoglycaemic events were not captured at the selected operating threshold. This reflects an intentional trade-off prioritising early detection while maintaining a manageable alert burden, which is essential to reduce alarm fatigue in real-world use. Importantly, the proposed model is not intended to replace existing CGM threshold-based alarms or closed-loop safety mechanisms, but rather to complement them by providing earlier risk estimates prior to reactive alerts. In practice, missed events may still be detected by standard CGM alarms, while the model contributes additional anticipatory information to support preventive action.

#### 4.1.6. Strengths of the Study

This study has several methodological strengths that enhance the credibility of its findings. First, all models were evaluated using patient-wise, leak-free train–validation–test splits, reducing the risk of optimistic bias arising from within-subject correlation. Second, performance was assessed using metrics appropriate for rare-event prediction, including precision–recall analysis and explicit probability calibration, rather than relying on ROC AUC alone. Third, cross-phase evaluation between Ramadan and the post-fasting period provided a realistic stress test under physiological and behavioural shift. Finally, the integration of multimodal CGM and wearable data, together with transparent calibration and interpretability analyses, supports both the robustness and clinical plausibility of the proposed framework.

#### 4.1.7. Model Selection Aligned with Clinical Use

Model selection was guided by clinical priorities rather than discrimination metrics alone. Because missed hypoglycaemic events pose greater risk than false alarms, we prioritised models that maintained high recall on the naturally imbalanced test set while exhibiting acceptable calibration, alert burden, and stability across fasting phases. Within this framework, a multimodal BiLSTM trained on Ramadan data emerges as a strong candidate for a prototype decision-support system, with operating thresholds in the 0.40–0.45 range tuned to balance sensitivity and alert volume. The all-features LSTM 50 and wearable-enhanced BiLSTM provide complementary configurations, enabling flexibility across varying levels of contextual and behavioural information while maintaining CGM as the core physiological signal.

More broadly, these findings suggest that deployment-ready hypoglycaemia prediction models should be evaluated using a combined profile of rare-event retrieval, alert burden, calibration, and robustness across behavioural states, rather than relying on ROC AUC alone. Accordingly, operating thresholds were selected to reflect clinically meaningful trade-offs rather than purely statistical optimisation, treating threshold selection as a clinical decision-support problem aligned with real-world safety and usability requirements.

#### 4.1.8. Temporal Modelling and Clinical Relevance of Hypoglycaemia Risk Prediction

The results demonstrate that incorporating temporal dynamics and Ramadan-specific behavioural proxy features substantially improves hypoglycaemia prediction performance without requiring aggressive class rebalancing. The strong performance observed in models trained on the original imbalanced dataset suggests that temporally structured features provide sufficient discriminatory information to partially mitigate the effects of class imbalance.

Although undersampling achieved higher F1-scores in certain configurations, this improvement primarily reflects a more balanced class distribution rather than genuine generalisation capability. In contrast, models trained without resampling exhibited more consistent performance across validation and test sets, indicating greater robustness and stability.

The observed discrepancy between the selected model (BiLSTM) and the best-performing model on the test set (LSTM 100) underscores the importance of strict validation-based model selection. Maintaining this distinction ensures an unbiased evaluation protocol and prevents implicit overfitting to the test set.

Notably, performance improved substantially at the daily aggregation level, indicating that hypoglycaemia risk is more effectively captured as a temporal process rather than as isolated hourly events. This finding highlights the clinical relevance of aggregated risk prediction for decision-making during Ramadan fasting.

### 4.2. Comparison with Prior Work

Most previous hypoglycaemia prediction studies have focused on narrow or highly structured contexts, such as nocturnal periods, postprandial windows, or inpatient care, and have relied primarily on continuous glucose monitoring (CGM) or electronic health record data. In outpatient settings, CGM-based nocturnal models typically report ROC AUC values in the range of 0.79–0.84 with sensitivities of approximately 70–75%, while postprandial and inpatient models may achieve higher ROC AUCs (0.89–0.97) but only within restricted physiological periods or using dense hospital data that are not available in free-living contexts [15,16,17,18,19,20].

In contrast, the present study evaluates hour-ahead hypoglycaemia prediction continuously across the full 24 h day in free-living adults with type 1 diabetes during Ramadan, using rolling 36 h sequences and operating under a natural prevalence of approximately 4% hypoglycaemic hours. Under these demanding conditions, the multimodal LSTM achieved an ROC AUC of 0.867 and a PR AUC of 0.341 on the imbalanced test set, with recall of 0.77. When assessed on a balanced diagnostic subset, precision–recall performance increased substantially, indicating that the underlying discriminative ability is comparable to that of state-of-the-art models once prevalence effects are removed.

A key distinction of this work lies in its behaviour-aware multimodal design. Whereas prior wearable-based studies have largely focused on passive detection of hypoglycaemia [21,36], our results demonstrate that wearable-derived activity, cardiovascular physiology, and sleep signals can support proactive hour-ahead forecasting. Notably, a wearable BiLSTM achieved an ROC AUC of 0.915 and a PR AUC of 0.522, approaching or exceeding the performance of CGM-only baselines. This finding extends earlier smartwatch-based detection studies from retrospective identification toward anticipatory risk estimation.

Ramadan-specific predictive research remains sparse and has focused almost exclusively on adults with type 2 diabetes. The PROFAST–IT study demonstrated limited hypoglycaemia prediction during fasting despite strong performance for other glycaemic outcomes [24]. In contrast, the present study targets adults with type 1 diabetes and integrates CGM with wearable physiology, sleep, and visit-level therapy data, explicitly modelling fasting-related behavioural and circadian disruption. To the best of our knowledge, this represents the first behaviour-aware, multimodal framework to provide calibrated, hour-ahead hypoglycaemia risk prediction in fasting adults with type 1 diabetes under real-world class imbalance.

More broadly, our work reframes hypoglycaemia prediction during Ramadan from a glucose-only ranking task to a calibrated, behaviour-aware early-warning problem suitable for real-world clinical decision support.

While the proposed model leverages temporal dynamics and behavioural proxies to capture circadian patterns, it does not explicitly incorporate environmental drivers such as ambient light exposure. This represents an important limitation but also a clear opportunity for future improvement. Emerging evidence indicates that light exposure is a primary regulator of circadian physiology and plays a direct role in metabolic control. Experimental studies have demonstrated that increased exposure to natural daylight enhances glycaemic stability and metabolic flexibility [25], while epidemiological analyses using wearable light sensors have linked nighttime light exposure to increased diabetes risk [26]. These findings suggest that integrating continuous light exposure measurements could further improve both predictive performance and physiological interpretability.

### 4.3. Limitations

Despite the promising performance of the proposed temporal and Ramadan-aware hypoglycaemia prediction framework, several limitations warrant consideration.

First, the dataset was relatively small and markedly imbalanced, with hypoglycaemic events comprising approximately 4–5% of observations, while this reflects real-world clinical distributions, the combination of limited sample size and event rarity may constrain model capacity, reduce recall, and contribute to wider uncertainty in performance estimates. Although multiple resampling strategies were explored, these often degraded the precision–recall balance, suggesting that future work should focus on more advanced imbalance-aware learning approaches, such as cost-sensitive loss functions or focal loss.

Second, wearable data completeness was moderate (approximately 50–70%) and exhibited partially systematic missingness, particularly affecting sleep-related signals due to off-wrist periods during nocturnal hours, while the adopted approach allowed the model to learn directly from incomplete observations without introducing artificial temporal structure, reduced nocturnal coverage may have attenuated the contribution of sleep-related features and represents a potential source of physiological bias.

The absence of temporal imputation strategies reflects a deliberate design choice to preserve a realistic representation of real-world wearable and CGM data conditions, while this enhances ecological validity, it may introduce variability and contribute to missed hypoglycaemic events. Although CGM features were derived using within-hour aggregation (e.g., mean and standard deviation), no values were imputed across time to reconstruct missing observations. Future work should explore advanced missing-data modelling strategies to improve robustness while maintaining physiological validity.

Wearable-derived SpO_2_ data were treated as contextual indices rather than clinically validated physiological measurements. These signals may be affected by device-specific variability, signal noise, and context-dependent accuracy, particularly in free-living conditions. In addition, SpO_2_ exhibited substantial missingness (approximately 70%), reflecting real-world wearable usage patterns. Accordingly, SpO_2_ was not used for clinical interpretation or diagnostic inference, but instead contributed as a complementary contextual feature alongside other behavioural and physiological signals (e.g., heart rate, activity, and sleep), while this approach enhances ecological validity, it also represents a limitation, and future work should incorporate clinically validated measurements where available.

Third, the study cohort was relatively small (n=33), young, and clinically homogeneous, with all participants monitored using a single closed-loop insulin delivery system and a single wearable device, while this controlled configuration reduced technical variability and enabled consistent multimodal integration, it may limit generalisability to broader populations, diverse clinical settings, and heterogeneous device ecosystems. Inter-device variability, differences in sensor accuracy, and variability in data availability may influence model performance in real-world deployments. Accordingly, future work should prioritise validation across larger, multi-centre cohorts and diverse device platforms.

Fourth, although rich temporal and behavioural proxy features were incorporated to approximate circadian dynamics, direct environmental signals such as ambient light exposure were not available. Given the established role of light in circadian regulation and glucose metabolism, future work should integrate wearable-based light sensing modalities, including intensity, timing, and spectral characteristics, to enable more physiologically grounded modelling.

Fifth, the current framework focuses on hourly and daily prediction horizons; while daily aggregation improved clinical interpretability and predictive stability, the model does not yet support real-time adaptive prediction or individualised early-warning horizons. Future research should explore personalised prediction windows, online learning strategies, and patient-specific calibration.

Finally, the proposed approach relies on deep learning architectures that, while effective, may limit interpretability for clinical decision-making. Incorporating explainability techniques, such as attention mechanisms, feature attribution methods, or interpretable temporal models, will be essential to enhance transparency and facilitate clinical adoption.

Overall, future work should focus on improving data completeness, integrating multimodal environmental sensing, validating across diverse populations, and advancing toward personalised, real-time, and interpretable prediction systems to support clinically robust hypoglycaemia risk assessment in real-world settings.

### 4.4. Implications and Future Directions

The primary implication of this work is that hour-ahead hypoglycaemia forecasting can be operationalised as a practical early-warning capability rather than a retrospective risk-ranking task. Rather than identifying a single universally optimal architecture, our findings support a deployment-oriented framework in which forecasting performance, calibration, alert stability, and robustness across behavioural states are jointly optimised. Conceptually, this pathway progresses from retrospective evaluation, to prospective “silent” deployment, to threshold-tuned user-facing alerts, and finally to integration within decision-support or closed-loop systems.

In a prototype decision-support system, a multimodal BiLSTM trained on Ramadan data would serve as the primary forecasting engine, ingesting harmonised CGM and wearable streams and generating rolling one-step-ahead risk estimates at hourly resolution. Operating thresholds could be tuned within a clinically meaningful range (e.g., 0.40–0.45) to achieve high hypoglycaemia recall (e.g., ≥0.75) while constraining daily alert volume. Simple temporal smoothing strategies, such as N-of-M alert rules, can further suppress transient probability spikes and promote stable, interpretable alert behaviour. Importantly, wearable-enhanced BiLSTM configurations provide strong complementary performance by incorporating behavioural and physiological context alongside CGM signals, while compact CGM-focused LSTM variants offer stable baseline performance when additional contextual inputs are limited. Together, these configurations enable flexible deployment across settings with varying levels of contextual data availability, without compromising the central role of CGM as the primary physiological signal.

Before any clinical deployment, this forecasting pipeline should undergo external validation in independent cohorts, followed by a prospective “silent” evaluation phase in which risk scores are generated and logged without triggering user-facing alerts. Such a phase would enable calibration drift assessment, alert-volume characterisation, and threshold refinement under real-world conditions without influencing care. Iterative usability testing with clinicians and people with type 1 diabetes will be essential to optimise risk visualisation, alert timing, and recommended responses.

More broadly, the robustness of models trained under fasting conditions suggests that physiologically constrained states, such as Ramadan, may offer informative training regimes for generalisable short-horizon risk forecasting. Embedding hour-ahead risk models within mobile decision-support applications or closed-loop insulin delivery ecosystems could support proactive mitigation strategies, including personalised carbohydrate intake, insulin adjustment, and activity planning, while minimising alarm fatigue through calibrated and temporally stable predictions.

The proposed framework establishes a pathway toward real-time, temporally and behaviourally adaptive clinical decision support, enabling proactive identification and mitigation of hypoglycaemia risk under high-risk fasting conditions.

## 5. Conclusions

This study demonstrates that behaviour-aware, multimodal recurrent deep-learning models can provide calibrated, hour-ahead hypoglycaemia risk forecasts for free-living adults with type 1 diabetes during and after Ramadan fasting. By integrating CGM with wearable-derived activity, cardiovascular, sleep, visit-level, and static clinical information, the proposed framework advances beyond glucose-only prediction toward a more comprehensive representation of fasting-related physiology and behaviour.

Under severe real-world class imbalance (approximately 4% hypoglycaemic hours), the models achieved reliable rare-event detection, maintaining clinically meaningful sensitivity (approximately 77% recall for next-hour events) while preserving stable probability calibration and robustness across fasting and post-fasting phases. Importantly, explicit modelling of temporal dynamics and Ramadan-specific behavioural proxy features was a key driver of performance, enabling accurate hypoglycaemia forecasting without reliance on aggressive class rebalancing. This finding highlights the importance of preserving natural temporal structure in rare-event prediction settings.

Wearable-enhanced recurrent models approached, and in some configurations exceeded, the precision–recall performance of CGM-only baselines, demonstrating that behavioural and physiological context provides substantial complementary predictive signal beyond glucose dynamics. This enables robust performance across varying levels of contextual data availability while preserving CGM as the core physiological signal.

Taken together, these findings establish hour-ahead hypoglycaemia forecasting as a feasible and clinically actionable early-warning capability rather than a retrospective, alarm-driven risk-ranking task. This paradigm shift enables proactive risk mitigation—such as timely carbohydrate intake, insulin adjustment, or activity modification—before symptomatic or reactive alerts occur, while maintaining a clinically acceptable balance between event detection and alert burden.

The proposed framework provides a practical pathway toward deployment within decision-support systems and closed-loop diabetes technologies. Future work should prioritise external validation in diverse cohorts, prospective “silent-mode” evaluation to characterise real-world performance and alert burden, and the integration of personalised and adaptive prediction strategies to support safe, reliable, and clinically effective adoption.

## Figures and Tables

**Table 1:** Cohort characteristics and data yield after harmonisation and quality control. Baseline clinical characteristics and sensor data availability are summarised to describe the study population. Exploratory comparisons of selected variables in the 6 h preceding hypoglycaemia events are included to contextualise multimodal feature behaviour and were not used for inferential testing.

Variable/Metric	Mean ± SD/Value	Median [IQR]/6 h Comparison/Notes	Range (Min–Max)
**Participants**			
Total participants with CGM	33	Included if hourly aggregation was possible (hours with <4 valid CGM readings excluded)	–
Participants with baseline data	35	Clinical characteristics available	–
Average valid CGM hours	1274.7	Hours with ≥4 readings retained	–
Minimum hypoglycaemia hours	7	Per participant	–
Maximum hypoglycaemia hours	188	Per participant	–
Mean hypoglycaemia hours	59.1	Per participant	–
**Baseline characteristics (static and visit features;** ***N*** **= 35)**			
Age (years)	27.9 ± 8.4	25.0 [22.0–32.0]	18–49
BMI (kg/m^2^)	28.4 ± 5.9	28.6 [23.9–31.3]	17.3–43.4
HbA1c (%)	7.59 ± 1.22	7.35 [6.70–8.35]	5.40–10.10
Cholesterol (mmol/L)	4.80 ± 1.13	4.40 [4.03–5.65]	3.20–7.90
LDL (mmol/L)	2.77 ± 0.92	2.50 [2.20–3.35]	1.20–5.80
HDL (mmol/L)	1.61 ± 0.36	1.63 [1.27–1.85]	1.00–2.40
Triglycerides (mmol/L)	1.05 ± 0.93	0.80 [0.60–1.05]	0.40–5.60
eGFR (mL/min/1.73 m^2^)	86.9 ± 14.9	90.0 [90.0–90.0]	2.3–90.0
Creatinine (μmol/L)	68.9 ± 19.0	69.0 [53.5–78.0]	30–113
Insulin dose (U/kg/day)	0.75 ± 0.26	0.68 [0.58–0.85]	0.43–1.53
SmartGuard (%)	87.7 ± 17.0	97.0 [80.5–99.0]	38–100
Sex	–	F: 19 (54.3%); M: 16 (45.7%)	–
**CGM and wearable data yield (** ***N*** **= 33)**			
CGM days per participant	57.8 ± 8.5	–	41–74
Valid CGM hours	1274.7 ± 310	–	–
Hypoglycaemia hours	59.1 ± 48.2	–	7–188
CGM glucose (mg/dL)	–	161.15 → 138.17	–
Steps/day	2914 ± 2603	21.55 → 1212.12	0–7036
Distance/day (m)	1920 ± 1875	18.07 → 832.00	0–4828
Calories/day (kcal)	91.2 ± 68.9	0.676 → 41.775	0–203
Mean heart rate (bpm)	98.7 ± 26.4	126.99 → 119.03	63–250
**CGM and wearable data yield (** ***N*** **= 33)**			
Total sleep duration (min/day)	261 ± 112	–	0–425
Mean SpO_2_ (device index)	–	–	15–34
Awake ratio	–	0.095 → 0.331	–
Deep sleep ratio	–	0.651 → 0.766	–
Light sleep ratio	–	1.347 → 1.385	–
REM sleep ratio	–	0.411 → 0.474	–

*Abbreviations:* CGM = continuous glucose monitoring; HbA1c = glycated haemoglobin. *CGM inclusion rule:* Time-series analyses included participants with usable CGM coverage across Ramadan and the after-fasting month; hourly summaries excluded hours with fewer than four valid CGM readings. [a] Values show overall mean → mean within the 6 h preceding hypoglycaemia events. [b] *eGFR note:* Estimated glomerular filtration rate values reflect laboratory-reported measurements; lower-bound values correspond to isolated readings at or near the assay reporting limit rather than persistent severe renal impairment. [c] *SpO_2_ note:* SpO_2_ values represent a device-specific index reported by the wearable sensor and do not correspond directly to arterial oxygen saturation measured by clinical-grade oximetry.

**Table 2:** Dataset composition across splits.

Dataset Split	Total Sequences	Hypoglycaemia, n (%)	Non-Hypoglycaemia, n (%)
Training	12,870	604 (4.7%)	12,266 (95.3%)
Validation	1991	80 (4.0%)	1911 (96.0%)
Test	3638	133 (3.7%)	3505 (96.3%)
Total	18,499	817 (4.4%)	17,682 (95.6%)

**Table 3:** Performance metrics for evaluated models (original training and test sets).

Group	Model	Thr	Acc	F1w	Prec1	Rec1	F11	Spec1	ROC AUC	PR AUC	Brier
CGM + Temporal + Wear + Static	LSTM 100	0.40	**0.966**	0.468	**0.539**	0.414	–	0.987	**0.909**	**0.454**	**0.025**
CGM + Temporal + Wear + Static	LSTM 50	0.40	**0.965**	0.423	0.528	0.353	–	**0.988**	0.904	0.416	0.026
CGM + Temporal + Wear + Static	BiLSTM	0.40	**0.964**	0.423	0.511	0.361	–	0.987	0.897	0.390	0.027
All	LSTM 50	0.58	0.885	0.914	0.195	0.692	0.305	0.892	0.867	0.341	0.100
All	LSTM 50	0.60	0.889	0.916	0.198	0.669	0.306	0.897	0.867	0.341	0.100
All	BiLSTM	0.42	0.901	0.924	0.209	0.609	**0.311**	0.912	0.847	0.306	**0.065**
All	BiLSTM	0.40	0.899	0.922	0.204	0.609	0.306	0.910	0.847	0.306	**0.065**
All	BiLSTM	0.50	**0.912**	**0.929**	**0.219**	0.549	**0.313**	**0.926**	0.847	0.306	**0.065**
CGM + Wear + Static	LSTM 50	0.60	0.904	0.925	0.221	0.647	0.329	0.913	0.874	0.222	0.085
CGM + Wear + Static	LSTM 100	0.40	0.916	0.932	0.226	0.534	0.318	0.931	0.856	0.294	0.058
CGM + Wear + Static	LSTM 100	0.46	0.921	0.935	0.234	0.511	0.321	0.936	0.856	0.294	0.058
CGM + Wear + Static	LSTM 100	0.50	0.924	0.936	0.241	0.504	0.326	0.940	0.856	0.294	0.058
CGM + Wear + Static	LSTM 50	0.40	**0.937**	**0.944**	**0.279**	0.459	**0.347**	**0.955**	0.852	0.312	**0.049**
CGM + Wear	BiLSTM	0.40	0.881	0.913	0.195	**0.791**	0.313	0.884	**0.915**	**0.522**	0.079
CGM + Wear	LSTM 25 L1	0.41	0.893	0.921	0.213	0.785	0.335	0.897	0.910	0.497	0.074
CGM + Wear	BiLSTM	0.45	0.902	0.927	0.229	0.780	0.354	0.906	**0.915**	**0.522**	0.079
CGM + Wear	LSTM 100	0.41	**0.907**	**0.930**	**0.237**	0.763	**0.361**	**0.912**	0.905	0.454	**0.059**
CGM + Wear	LSTM 100	0.40	0.904	0.928	0.230	0.763	0.354	0.909	0.905	0.454	**0.059**
CGM	LSTM 25 L2	0.41	**0.889**	**0.916**	0.206	**0.697**	**0.319**	**0.896**	0.868	0.346	**0.077**
CGM	LSTM 100	0.40	0.840	0.886	0.148	0.694	0.244	0.846	0.848	0.313	0.095
CGM	LSTM 50	0.40	0.872	0.906	**0.181**	0.691	0.286	0.879	0.857	0.324	0.079
CGM	BiLSTM	0.40	0.870	0.905	0.178	0.688	0.283	0.877	0.857	0.353	0.082
CGM	LSTM 25 L1	0.40	0.861	0.899	0.167	0.688	0.269	0.868	0.848	0.322	0.082

*Abbreviations:* **CGM + Temporal + Wear + Static** = CGM time-series features + wearable time-series features + Ramadan temporal variables + static baseline covariates; **All** = CGM time-series features + wearable time-series features + Ramadan visit-level variables + static baseline covariates; **CGM +Wear + Static** = CGM time-series features + wearable time-series features + static baseline covariates; **CGM +Wear** = CGM time-series features + wearable time-series features only; **CGM** = CGM time-series features only. **Note:** Bold values indicate the best performance within each feature group.

## Data Availability

De-identified participant data are available from the corresponding author upon reasonable request. Requests will be evaluated for scientific merit and are subject to approval through a data-use agreement. Data subject to institutional, ethical, or intellectual property restrictions cannot be shared.

## Abbreviations

The following abbreviations are used in this manuscript:

T1D	Type 1 diabetes
CGM	Continuous glucose monitoring
PR AUC	Area under the precision–recall curve
ROC AUC	Area under the receiver-operating characteristic curve
ECE	Expected calibration error
LSTM	Long short-term memory
BiLSTM	Bidirectional long short-term memory
AHCL	Advanced hybrid closed-loop

## Appendix A. Overview of Previous Studies

Table A1 summarises representative prior studies on hypoglycaemia prediction and detection across inpatient and free-living settings, including data modalities, modelling approaches, and study objectives. These studies predominantly rely on CGM, electronic health records, or wearable signals analysed in isolation using classical machine-learning and deep-learning methods. The present work extends this literature by explicitly integrating CGM with wearable-derived behavioural and physiological context for hour-ahead risk forecasting during Ramadan.

**Table A1:** Representative ML studies on hypoglycaemia prediction/detection (aim, cohort, inputs, methods).

Ref	Year	Aim of Research	Population/Setting	Inputs	Methods
[15]	2020	Bedtime prediction of nocturnal hypoglycaemia	Outpatient T1D	CGM features	LDA
[16]	2019	00:00–06:00 nocturnal hypoglycaemia prediction	Admissions (n=10,000)	CGM	Random forest
[17]	2019	Forecast postprandial hypoglycaemia risk	Adults with T1D	CGM + meal/insulin	RF; SVM
[18]	2019	Predict postprandial hypoglycaemia	Mixed T1D/T2D adults	CGM + meal context	Gradient boosting
[19]	2020	Predict inpatient hypoglycaemia using EHR data	Admissions (n=17,658)	EHR (labs, vitals, meds)	XGBoost; LR
[20]	2022	Short-horizon nocturnal prediction in hospitalized T1D	Hospitalized T1D (n=406)	CGM + clinical data	RF; LASSO-LR; ANN
[21]	2023	Non-invasive hypoglycaemia detection via smartwatch signals	Insulin-treated diabetes	HR/HRV/EDA + CGM	Tree-based ML; SHAP
[22]	2024	60-min hypo/hyper prediction with explainability	Outpatient T1D (n=153)	CGM + engineered features	XGBoost; SHAP
[24]	2020	Predict glucose variability during fasting	Fasting T2D cohort	CGM + Fitbit + EHR	XGBoost; SHAP
[37]	2020	Classify overnight glycaemic control	Outpatient T1D	CGM + meals/insulin	SVM; ANN
[38]	2018	ODE physiology with ANN glucose prediction	Pilot cohort	CGM + physiology	ANN + simulation
[39]	2018	ODE-based MPC to prevent dysglycaemia	Adults with T1D	CGM + insulin/meal timing	MPC
[40]	2014	Early ML for CGM-based glucose prediction	Mixed T1D/T2D	CGM only	NN; regression
[36]	2024	Daytime activity linked to nocturnal hypoglycaemia	Children with T1D	CGM + physical activity	RF; DNN

## Appendix B. Overview of Dataset Quality

Appendix B provides supplementary tables illustrating dataset structure, feature availability, and missingness patterns to support transparency and reproducibility. Detailed data preprocessing, harmonisation, and modelling decisions are described in the Methods (Section 2.2 and Section 2.4). All summaries were generated using the public analysis notebooks available in the project repository [41].

### Appendix B.1. Dataset Excerpts

Table A2 presents a short excerpt of synchronised hourly CGM and wearable data from a representative participant during Ramadan, illustrating the temporal alignment of multimodal signals. Table A3 summarises baseline clinical characteristics for a subset of participants to document cohort heterogeneity.

**Table A2:** Excerpt from the synchronised intraday dataset showing ten consecutive hourly records from a representative participant during Ramadan.

PatientID	Start	Period	Visit	Cgm	Steps	Calories	Distance	Heart_Rate	Spo2	Deep	Light	Rem	Nap	Awake
45	22 March 2023 00:00	Ramadan	V1	229.9	52	38.5	0.05	76	98	47	118	33	0	52
45	22 March 2023 01:00	Ramadan	V1	203.9	60	45.1	0.06	73	97	50	121	35	0	48
45	22 March 2023 02:00	Ramadan	V1	178.5	41	28.3	0.03	71	97	55	105	32	0	59
45	22 March 2023 03:00	Ramadan	V1	153.9	32	21.2	0.02	70	97	48	96	27	0	69
45	22 March 2023 04:00	Ramadan	V1	132.8	20	14.6	0.01	68	97	52	89	24	0	72
45	22 March 2023 05:00	Ramadan	V1	125.6	18	13.9	0.01	69	97	49	90	23	0	73
45	22 March 2023 06:00	Ramadan	V1	139.3	25	16.4	0.02	71	97	45	110	29	0	68
45	22 March 2023 07:00	Ramadan	V1	148.4	49	36.2	0.04	74	98	50	115	30	0	61
45	22 March 2023 08:00	Ramadan	V1	160.5	88	62.4	0.09	77	98	55	122	34	0	57
45	22 March 2023 09:00	Ramadan	V1	174.3	132	89.3	0.15	80	98	60	129	37	0	52

**Table A3:** Excerpt from the harmonised visit-level dataset showing the first ten participants’ baseline and visit-specific features during Ramadan.

Code	Age (y)	Gender	Duration (y)	Retinopathy	Nephropathy	Height (cm)	Weight (kg)	BMI	PatientID (Huawei)
R01	22	F	14	mild NPDR	albuminuria	149.0	78.1	35.2	45
R02	47	F	25	mild NPDR	no	159.0	61.3	24.9	46
R04	26	M	15	no	no	174.6	109.0	35.8	47
R05	19	M	15	no	albuminuria	155.0	76.0	31.6	48
R06	34	M	15	no	no	163.0	75.0	28.2	49
R07	25	F	20	no	no	157.0	73.7	29.9	53
R10	25	F	19	yes	no	171.0	81.4	27.8	54
R11	25	M	20	yes	albuminuria	165.0	66.4	24.4	55
R12	27	M	10	no	no	—	—	—	57
R15	22	F	10	yes	no	153.5	61.0	25.9	59

### Appendix B.2. Dataset Completeness and Quality Control

Feature-level missingness was quantified to document data availability across CGM, wearable-derived, and visit-level variables and to inform downstream quality-control decisions. Table A4 summarises missingness percentages across study phases for dynamic and clinical feature blocks.

**Table A4:** Summary of missingness across dynamic CGM/wearable features and the top twenty visit-level variables.

Feature	Category	Ramadan (%)	After-Fasting (%)	Overall (%)
**Dynamic CGM and wearable features**				
CGM readings	CGM	12.26	10.69	16.99
Steps	Wearable activity	45.3	49.0	47.1
Calories	Wearable activity	46.8	48.3	47.6
Heart rate	Wearable physiology	64.1	66.2	65.3
SpO_2_	Wearable physiology	70.5	71.6	71.0
Sleep stages	Wearable sleep	≈87	≈87	≈87
**Visit-level clinical and therapy variables (top 20)**				
Smartguard Exit main reason	Pump settings	42.70	–	39.52
SBP	Blood pressure	13.33	–	16.43
DBP	Blood pressure	13.33	–	16.43
HbA1C	Lab results	17.14	–	15.24
GMI	SmartGuard data	12.59	–	15.02
Cholesterol	Lab results	14.29	–	13.57
LDL	Lab results	14.29	–	13.57
HDL	Lab results	14.29	–	13.57
Triglyceride	Lab results	14.29	–	13.57
Hemoglobin	Lab results	12.59	–	12.94
Creatinine	Lab results	12.59	–	12.94
eGFR	Renal marker	12.59	–	12.94
BMI	Anthropometric	8.57	–	8.10
HbA1c (%)	Lab results	8.57	–	8.10
SmartGuard (%)	Device parameter	5.71	–	5.71
Carbohydrates (g/day)	Lifestyle/therapy	4.29	–	4.57
Meals/Day	Lifestyle/therapy	4.29	–	4.57
Total Daily Dose (U)	Insulin/device	4.29	–	4.57
Fasting (% of 29 days)	Adherence	2.86	–	2.86
SmartGuard Target	Device settings	0.00	–	1.43

### Appendix B.3. Missing Data Handling (TRIPOD-AI Compliant)

Missing data were observed across wearable-derived modalities, with overall data completeness ranging between 50 and 70%. Missingness primarily reflected real-world device usage patterns, including off-wrist periods, charging intervals, and user-dependent behaviours.

The missingness mechanism was therefore considered partially systematic, with a pronounced effect on sleep-related signals during nocturnal periods.

In accordance with TRIPOD-AI reporting guidelines, no temporal imputation strategies were applied to avoid introducing artificial physiological structure. Instead, missing values were retained and implicitly handled within the modelling pipeline. This design enables the model to leverage available temporal context while minimising bias associated with synthetic data reconstruction.

The potential impact of missingness on model performance and physiological interpretability is discussed in Section 2.2 and Section 4.

### Appendix B.4. Statistical Overview of the Wearable Dataset

Descriptive summaries of static, behavioural, and physiological variables were generated to contextualise missingness patterns across Ramadan and the post-fasting period. These summaries were reproduced from the public analysis notebooks (Analysis of HMC Model—Static; HMC Statistical Overview variables) [41] and confirm stable CGM integrity across study phases.

Distributions of dynamic wearable variables were mildly left-skewed, with physiologically plausible high-end values reflecting periods of increased activity or postprandial excursions (Figure A1a,b). These observations were retained to preserve realistic variability for model training.

Hourly CGM profiles across participants (Figure A1c,d) show consistent circadian structure, with early-morning glucose nadirs during prolonged fasting and higher evening values following iftar, supporting the internal consistency of the harmonised intraday time-series.

## Appendix C. Feature Diagnostics and Interpretability

This appendix provides supplementary diagnostics supporting the multimodal feature set and interpretability analyses used in the main Results (Section 3.1) and Methods (Section 2.2). We report feature grouping, correlation structure, redundancy checks (VIF), Random Forest importance, PCA summaries, and SHAP-based explanations. All analyses were reproduced from the public notebooks in the project repository [41].

### Appendix C.1. Overview of Features

We summarised the static, visit-level, and dynamic physiological variables, see Table A5, that form the backbone of the modelling pipeline. These features capture the stable clinical profile of each participant as well as the behavioural and metabolic variability that characterises fasting physiology during Ramadan. Baseline characteristics—BMI, HbA1c, lipids, renal markers, insulin dose, and SmartGuard%—showed clinically coherent relationships. As shown in Figure A5a,b, cholesterol and LDL were tightly positively correlated, while creatinine and eGFR showed their expected inverse relationship. HDL and triglycerides showed a mild inverse trend, and BMI had only weak association with HbA1c, indicating that glucose control in this cohort was shaped more by therapy and behaviour than by body size. No problematic multicollinearity was detected (all |r|<0.8), supporting the retention of the full static block.

### Appendix C.2. Feature Organisation

Table A5 summarises the feature blocks used in the harmonised CGM–wearable dataset, including dynamic, visit-level, static baseline, and contextual variables, together with PCA-derived components used for modelling.

### Appendix C.3. Correlation Structure, Redundancy, and Dimensionality Reduction

Correlation patterns and feature rankings are provided in Figure A2. Redundancy was assessed using VIF, and all features showed acceptable collinearity (VIF <5), with expected clustering among activity variables. Random Forest importance rankings were used as an interpretable benchmark and were consistent across Ramadan and the post-fasting period. Full VIF values, importance scores, and PCA loadings are reported in Table A6.

**Table A5:** Feature blocks, principal components, and sequence-model inputs used in the harmonised CGM–wearable dataset.

**Feature Block**	**Variables Included**		**Purpose**
**Dynamic (time-varying)**	cgm_min, cgm_max, cgm_mean, cgm_std, steps, calories, distance, deep, light, rem, nap, awake, heart_rate, spo2		Captures hour-to-hour physiological and behavioural changes.
**Visit-level**	carb, meals, total_daily_dose_u, fasting_pct_29		Summarises short-term lifestyle, insulin therapy, and fasting adherence.
**Static baseline**	BMI, HbA1c, LDL, HDL, triglycerides, eGFR, creatinine, SmartGuard_percent		Stable metabolic and clinical markers used for individualisation.
**Contextual metadata**	hour_of_day, visit_assigned, period_main		Aligns each time point to Ramadan or after-fasting study phases.
**Outcome**	hypo_event_next_hour		Binary label for hour-ahead hypoglycaemia.
**Wearable Feature**	**Included Variables**		**Purpose**
CGM statistics	cgm_min, cgm_max, cgm_mean, cgm_std, cgm_mean ± std		Summarises hourly glucose level and variability.
CGM statistics (PCA)	3 components (74.2%/25.7%/0.1%)		Amplitude and variability axes
CGM PCA components	pca_cgm1, pca_cgm2, pca_cgm3		Latent glycaemic dynamics (PCA).
Wearable PCA components	pc1_activity_energy, pc2_physiology, pc3_sleep_rest		Compressed lifestyle and physiology representations.
**Block**	**n Components**	**Explained Variance**	**Interpretation**
CGM statistics	3	95.7%/4.1%/0.1%	Amplitude and variability axes
Lifestyle (wearable)	3	88.3%/9.0%/1.7%	PC1: activity/energy; PC2: physiology; PC3: sleep/rest

**Table A6:** Summary of multicollinearity (VIF), Random Forest importance, and PCA loadings for lifestyle and physiological features. The first three components explain 98.5% of total variance.

Feature	VIF	RF Importance (%)	PC1	PC2/PC3
steps	4.99	17.13	0.167	0.883/−0.007
distance	2.99	11.03	0.087	0.429/0.017
calories	2.70	26.78	0.008	0.027/−0.002
heart_rate	1.26	29.35	0.982	−0.188/−0.027
spo2	1.17	7.68	0.026	−0.006/0.999
sleep-light	1.07	2.61	−0.001	−0.001/0.006
sleep-rem	1.04	2.10	−0.000	−0.000/0.003
sleep-deep	1.04	1.26	−0.001	−0.000/0.004
nap	1.01	0.84	−0.000	−0.000/0.002
awake	1.01	1.22	0.000	0.000/0.001
**Cumulative PCA variance:** [0.7544, 0.9334, 0.9852]				

#### Principal Component Structure

PCA was used to obtain compact representations of CGM and wearable blocks. For CGM, three components explained 74.2%, 25.7%, and 0.1% of variance; for lifestyle variables, three components explained 88.3%, 9.0%, and 1.7% during Ramadan. Conceptual interpretations of components are illustrated in Figure A3 and summarised in Table A5.

To evaluate the contribution of low-variance components, we re-trained the primary models after excluding the third PCA component from CGM and wearable feature blocks. Across all evaluated configurations, performance metrics remained stable, with negligible differences in ROC AUC (<0.01), PR AUC (<0.01), and recall (<0.02). These findings confirm that the third component does not materially influence predictive performance and was retained primarily for structural consistency and interpretability.

### Appendix C.4. Static and Visit-Level Feature Summaries

Additional static baseline relationships and visit-level feature dependencies are shown in Figure A4 and Figure A5, respectively, as supplementary support for the feature blocks used in modelling (Table A5). These summaries were reproduced from the public analysis notebooks [41].

Performance was evaluated using $R^{2}$ and RMSE on held-out data. Across models, bolus%, auto-basal%, and total daily insulin dose emerged as the strongest determinants of hypoglycaemia exposure, followed by meals/day, carbohydrate intake, and fasting%. Feature-importance patterns are shown in Figure A5b.

### Appendix C.5. Explainability

SHAP analyses were computed for the leak-safe multimodal BiLSTM on a subset of test sequences (GradientExplainer with DeepExplainer fallback where applicable). Global SHAP summaries for the sequence and static branches, together with a patient-level risk trajectory and per-window SHAP heatmap (patient 57), are shown in Figure A6.

## Appendix D. Our Pipeline Overview

This appendix provides supplementary implementation and evaluation details for the modelling pipeline described in Section 2.2, Section 2.3, Section 2.4, Section 2.5 and Section 2.6. We summarise the model architectures tested, the best-performing dual-input LSTM configuration, and extended threshold- and resampling-based performance diagnostics, including balanced diagnostic evaluation, cross-phase generalisation, and bootstrap uncertainty analyses. Code and analysis notebooks are available in the project repository [41].

The modelling pipeline followed a structured but flexible workflow designed to ensure transparency and reproducibility. All steps were implemented in Python 3.11 using standard open-source libraries with fixed random seeds for reproducibility. The process began with data integration and harmonisation, where CGM and wearable streams were aligned using ISO-standard timestamps and unified participant identifiers.

Model development was divided into two broad families. Classical learners—logistic regression, Random Forest, and XGBoost—served as baselines trained on aggregated non-sequential inputs. The primary models, however, were recurrent sequence architectures, including GRU, LSTM, BiLSTM, and the regularised LSTM 25 variants (L1 and L2). All recurrent networks consisted of two stacked LSTM-based layers followed by a ReLU dense projection and a sigmoid output that estimated the probability of hypoglycaemia in the next hour. Training used focal loss to emphasise minority-class samples, with α=0.25 and γ=2.0, and adaptive class weighting based on label frequencies. Optimisation relied on the Adam optimiser with a learning rate of $10^{-3}$ and early stopping based on validation loss to prevent overfitting.

A summary of all models evaluated, including their architecture depth, regularisation schemes, and intended role in this study, is provided in Table A7. Across the full set of experiments, the strongest performance was achieved using a dual-input LSTM architecture. This model combined temporal sequences of CGM-derived and wearable-derived features with static demographic and clinical covariates, allowing the network to integrate both individual context and short-term physiological dynamics. The fused representation produced well calibrated probability estimates for hour ahead hypoglycaemia and demonstrated the most robust behaviour across fasting and non-fasting phases.

**Table A7:** Summary of model architectures (CGM and multimodal experiments).

Model	Type	Depth	Reg.	Notes
LSTM 100	Deep LSTM	100 → 50	None	Reference high-capacity model
LSTM 50	Medium LSTM	50 → 25	None	Efficient, stable
LSTM 25(L1)	Sparse LSTM	50 → 25	L1	Sparsity, reduced AUC
LSTM 25(L2)	Smooth LSTM	50 → 25	L2	Best calibration
BiLSTM	Bidirectional	64 → 32	None	Highest PR AUC
ESN_MLP	Feed-forward	—	Dropout	Non-recurrent baseline
ESN_LSTM	Hybrid	32	Dropout	Lightweight sequential
DeepESN	Deep hybrid	64 → 32 → 16 → 32	Dropout	Stable, lower recall

### Appendix D.1. Best-Performing Model

The best-performing configuration was a dual-input LSTM combining 36 h temporal sequences with a static covariate branch. Table A8 reports the layer-wise architecture used for the final model.

**Table A8:** Layer-wise configuration of the best-performing dual-input LSTM.

Layer	Output Shape	Parameters
Sequence input	(None, 36, 8)	0
LSTM 100	(None, 3, 100)	43,600
LSTM 50	(None, 50)	30,200
Static input	(None, 12)	0
Dense (32 → 25)	(None, 25)	1691
Concatenate	(None, 75)	0
Dense (32 → 1), sigmoid	(None, 1)	1889
**Total trainable**	**77,380**	

**Table A9:** Threshold sensitivity analysis for the balanced diagnostic test set across all feature configurations and model architectures.

Experiment	Model	Thr	Acc	F1_w_	Prec_1_	Rec_1_	F1_1_	Spec_1_	ROC AUC	PR AUC	Brier
CGM + Temporal + static + wear	BiLSTM	0.40	0.7669	0.7646	0.7619	0.7218	0.7413	0.8120	0.8784	0.8703	0.286
CGM + Temporal + static + wear	LSTM 100	0.40	0.7895	0.7882	0.7857	0.7632	0.7743	0.8120	0.8963	0.9054	0.265
CGM + Temporal + static + wear	LSTM 50	0.40	0.7594	0.7580	0.7541	0.7143	0.7337	0.8045	0.8865	0.8930	0.285
All features	LSTM 50	0.49	0.7932	0.7927	0.8250	0.7444	0.7826	0.8421	0.8567	0.8609	0.15
All features	LSTM 50	0.50	0.7932	0.7927	0.8250	0.7444	0.7826	0.8421	0.8567	0.8609	0.15
All features	BiLSTM	0.40	0.8623	0.8617	0.8120	0.9188	0.8614	0.8077	0.9075	0.9161	0.15
All features	BiLSTM	0.42	0.8623	0.8617	0.8068	0.9271	0.8614	0.8018	0.9075	0.9161	0.15
All features	BiLSTM	0.50	0.8146	0.8140	0.7328	0.9165	0.8157	0.7143	0.8593	0.8777	0.17
Static + Wear + CGM	LSTM 50	0.40	0.8052	0.8052	0.7407	0.8844	0.8079	0.7331	0.8567	0.8479	0.16
Static + Wear + CGM	LSTM 50	0.50	0.8146	0.8141	0.7722	0.8617	0.8147	0.7594	0.8567	0.8479	0.16
Static + Wear + CGM	LSTM 50	0.60	0.8009	0.8008	0.8056	0.7940	0.7991	0.8165	0.8567	0.8479	0.16
Static + Wear + CGM	LSTM 100	0.50	0.8265	0.8264	0.7586	0.9071	0.8214	0.7519	0.8539	0.8704	0.16
Static + Wear + CGM	LSTM 100	0.60	0.8133	0.8133	0.7885	0.8335	0.8061	0.8421	0.8539	0.8704	0.16
Wear + CGM	BiLSTM	0.40	0.7888	0.7883	0.6667	0.9468	0.7833	0.6471	0.8507	0.8607	0.16
Wear + CGM	BiLSTM	0.46	0.8009	0.8008	0.7069	0.9053	0.7953	0.7060	0.8507	0.8607	0.16
Wear + CGM	BiLSTM	0.50	0.8009	0.8008	0.7069	0.9053	0.7953	0.7060	0.8507	0.8607	0.16
Wear + CGM	BiLSTM	0.60	0.7932	0.7932	0.7340	0.8417	0.7831	0.7594	0.8507	0.8607	0.16
CGM only	LSTM 25 L2	0.40	0.7662	0.7661	0.6596	0.9113	0.7668	0.6471	0.8479	0.8539	0.17
CGM only	LSTM 25 L2	0.46	0.7738	0.7737	0.7069	0.8586	0.7802	0.6996	0.8479	0.8539	0.17
CGM only	LSTM 25 L2	0.50	0.7738	0.7737	0.7069	0.8586	0.7802	0.6996	0.8479	0.8539	0.17
CGM only	LSTM 25 L2	0.60	0.7662	0.7661	0.7344	0.8192	0.7700	0.7594	0.8479	0.8539	0.17

This table reports threshold-sensitivity results on the 1:1 balanced diagnostic subset across feature configurations and recurrent architectures, including discrimination (ROC/PR AUC), class-wise metrics, and calibration (Brier score). This table supports the threshold-level trends summarised in the main Results. **Note:** All rows derive from the balanced diagnostic test set. Repeated rows (24–39) in the original CSV were removed for clarity. Performance metrics include accuracy, weighted F1, precision, recall, F1 for the minority class, specificity, ROC AUC, PR AUC, and Brier score across thresholds. Wear means wearable data.

**Table A10:** Performance of resampling configurations on the original diagnostic test set.

Experiment	Model	Method	Thr	Acc	F1_w_	Prec_1_	Rec_1_	F1_1_	Spec_1_	ROC AUC	PR AUC	Brier
**Static + temporal + wearable + CGM**												
Temporal + other	LSTM 100	oversample seq	0.60	0.931	0.395	0.304	0.563	NAN	0.946	0.851	0.377	0.06
Temporal + other	BiLSTM	oversample seq	0.60	0.928	0.388	0.298	0.550	NAN	0.944	0.846	0.365	0.06
Temporal + other	LSTM 50	undersample_seq	0.50	0.905	0.360	0.620	0.259	NAN	0.982	0.872	0.341	0.05
**Static + visit + wearable + CGM**												
All features	LSTM 50	oversample seq	0.50	0.8600	0.8990	0.1725	0.7443	0.2801	0.8645	0.8672	0.3405	0.09
All features	LSTM 50	oversample seq	0.58	0.8845	0.9139	0.1953	0.6917	0.3046	0.8918	0.8672	0.3405	0.09
All features	LSTM 50	oversample seq	0.60	0.8889	0.9164	0.1982	0.6692	0.3058	0.8973	0.8672	0.3405	0.09
All features	BiLSTM	smote	0.42	0.9013	0.9236	0.2087	0.6090	0.3109	0.9124	0.8466	0.3061	0.06
All features	BiLSTM	smote	0.50	0.9118	0.9295	0.2185	0.5488	0.3126	0.9255	0.8466	0.3061	0.06
**Static + wearable + CGM**												
CGM + Static + wearable	LSTM 50	undersample_seq	0.50	0.8776	0.9096	0.1855	0.6917	0.2925	0.8847	0.8744	0.2219	0.08
CGM + Static + wearable	LSTM 50	undersample_seq	0.60	0.9035	0.9253	0.2205	0.6466	0.3288	0.9132	0.8744	0.2219	0.08
CGM + Static + wearable	LSTM 100	oversample seq	0.46	0.9208	0.9346	0.2337	0.5113	0.3207	0.9363	0.8558	0.2944	0.05
CGM + Static + wearable	LSTM 100	oversample seq	0.50	0.9238	0.9364	0.2410	0.5037	0.3260	0.9398	0.8558	0.2944	0.05
CGM + Static + wearable	LSTM 50	oversample seq	0.40	0.9367	0.9441	0.2785	0.4586	0.3466	0.9549	0.8515	0.3122	0.04
**wearable + CGM**												
CGM + wearable	LSTM 25 L1	oversample seq	0.41	0.8929	0.9209	0.2132	0.7853	0.3353	0.8968	0.9098	0.4967	0.07
CGM + wearable	BiLSTM	oversample seq	0.45	0.9020	0.9266	0.2289	0.7796	0.3538	0.9064	0.9147	0.5220	0.07
CGM + wearable	LSTM 100	smoteenn	0.40	0.8774	0.9111	0.1890	0.7796	0.3043	0.8809	0.9053	0.4380	0.07
CGM + wearable	LSTM 100	oversample seq	0.40	0.9015	0.9261	0.2259	0.7683	0.3491	0.9062	0.9145	0.4928	0.06
CGM + wearable	LSTM 100	smote	0.40	0.9042	0.9278	0.2304	0.7627	0.3538	0.9092	0.9049	0.4542	0.05
**CGM only**												
CGM only	LSTM 25 L2	undersample_seq	0.41	0.8889	0.9164	0.2064	0.6974	0.3185	0.8963	0.8681	0.3457	0.07
CGM only	LSTM 100	smotetomek	0.40	0.8400	0.8857	0.1480	0.6940	0.2440	0.8456	0.8483	0.3128	0.09
CGM only	LSTM 50	smotetomek	0.41	0.8755	0.9079	0.1848	0.6875	0.2912	0.8827	0.8568	0.3241	0.07
CGM only	BiLSTM	oversample seq	0.40	0.8702	0.9046	0.1780	0.6875	0.2828	0.8773	0.8572	0.3533	0.08
CGM only	LSTM 25 L1	smoteenn	0.40	0.8608	0.8988	0.1670	0.6875	0.2688	0.8675	0.8482	0.3221	0.08

**Note:** All rows correspond to the original test set. Underscores are escaped for LaTeX. ROC AUC and PR AUC fields corrected for encoding.

Table A11 reports bootstrap-based pairwise ROC AUC comparisons (2000 replicates).

**Table A11:** Pairwise ROC AUC significance testing (bootstrap, n=2000).

Comparison	ΔAUC	SE	Z	*p*-Value	Interpretation
none/LSTM 100 vs. oversample seq/LSTM 100	+0.012	0.0075	1.64	0.10	Not significant
none/LSTM 100 vs. undersample_seq/BiLSTM	**+0.037**	0.0094	3.92	**<0.001**	Undersampling reduced AUC
oversample seq/LSTM 100 vs. undersample_seq/BiLSTM	+0.024	0.0117	2.09	**0.037**	Oversampling modestly higher

#### Appendix D.1.1. Detailed Cross-Phase Performance Metrics

Table A12 reports cross-phase generalisation between Ramadan and the post-fasting period for recurrent models, including discrimination and calibration metrics.

**Table A12:** Full cross-phase generalisation results between fasting (Ramadan) and post-fasting (Shawwal) periods. Best results in bold.

Direction	Model	Thr.	ROC AUC	PR AUC	F1_*w*_	Accuracy	Prec_1_	Brier
Ramadan → Shawwal	**BiLSTM**	0.40	**0.917**	**0.501**	0.960	**0.382**	0.627	**0.0286**
Ramadan → Shawwal	LSTM 100	0.41	0.908	0.465	0.958	0.342	0.609	0.0297
Ramadan → Shawwal	LSTM 25 L2	0.40	0.904	0.457	0.959	0.393	0.590	0.0301
Ramadan → Shawwal	LSTM 50	0.40	0.904	0.456	0.958	0.386	0.575	0.0300
Shawwal → Ramadan	BiLSTM	0.40	0.911	0.481	0.958	0.346	0.617	0.0295

#### Appendix D.1.2. Daily Risk Summaries for Case-Study Patients

Table A13 provides illustrative daily summaries for two patients, reporting evaluated windows, observed positives, maximum predicted risk, and prevalence.

**Table A13:** Condensed daily risk summary for two case-study patients.

PatientID	Date	$n_{\mathrm{windows}}$	True Positives	Risk Max	Prevalence
57	23 March 2023	22	0	0.1460724	0
57	27 March 2023	22	4	0.2526547	0.181818182
57	28 March 2023	24	3	0.31044775	0.125
57	6 April 2023	24	1	0.23302013	0.041666667
57	11 April 2023	24	3	0.07706769	0.125
69	24 March 2023	21	1	0.31618590	0.047619048
69	1 April 2023	24	2	0.31777490	0.083333333
69	3 April 2023	24	3	0.25921425	0.125
69	16 April 2023	24	5	0.33482254	0.208333333
69	18 April 2023	24	6	0.29631174	0.25

### Appendix D.2. Model Selection and Deployment Framework

We shortlisted deployment candidates from a broad set of recurrent architectures by prioritising clinical safety and operational feasibility rather than maximising ROC AUC alone. The primary objective was high sensitivity for hypoglycaemia (minimising missed events), while constraining alert burden to a tolerable level during Ramadan. Additional requirements included robust calibration, stability across Ramadan and the post-fasting month, and graceful degradation under intermittent wearable missingness.

Models (GRU/LSTM/BiLSTM and regularised LSTM25 variants) were evaluated across CGM-only, CGM + wearable, CGM + static + wearable, and full multimodal feature sets, and under multiple imbalance-handling strategies. Candidates were removed if they failed to outperform non-sequential baselines, showed unstable uncertainty, poor calibration, or very low hypoglycaemia-class recall under the imbalanced test distribution. The final shortlist consisted of: (i) an all-features LSTM 50 trained with sequence-level oversampling, (ii) a wearable BiLSTM, and (iii) a multimodal BiLSTM with the strongest cross-phase performance.

Selection used a compact scorecard covering rare-event performance under natural prevalence (emphasising PR AUC and hypoglycaemia-class recall), calibration (Brier score and reliability curves; Appendix E), robustness under cross-phase transfer, and alert burden estimated from confusion matrices via (TP+FP) alerts per hour/day. Thresholds were tuned on validation data (typically 0.40–0.60), and temporal smoothing (N-of-M rules) was explored to suppress isolated probability spikes and improve alert stability.

For deployment, we propose a compact model package: a multimodal BiLSTM as the primary model (selected for robust cross-phase behaviour and calibration), a wearable-enhanced BiLSTM that incorporates additional behavioural and physiological context, and a compact CGM-focused LSTM_25_(L2) providing a stable baseline when contextual inputs are limited. Together, these configurations support flexible deployment across settings with varying availability of contextual data, while maintaining CGM as the central physiological signal. These models should be treated as prototypes pending external validation, prospective “silent-mode” evaluation, and usability testing to refine thresholds, alert presentation, and response protocols before clinical use.

## Appendix E. Evaluation Metric Definitions

### Appendix E.1. Checklist for Appraising Hypoglycaemia Prediction Studies

When reviewing prior hypoglycaemia prediction work, we used a brief rubric covering: population/setting relevance; outcome definition and prediction horizon; data provenance and missing-data handling; leak-free design (patient-wise splits) and imbalance strategy; appropriateness of metrics (especially PR AUC, recall, and calibration); uncertainty reporting; generalisability across devices/cohorts; and code/method transparency. This rubric highlighted that the present study aligns well with best practice in multimodal modelling, calibration reporting, and leak-free evaluation, while remaining limited by modest sample size, single-centre recruitment, and device-specific data.

The performance of the model was evaluated under six resampling strategies (no, oversampling, undersampling, SMOTE, SMOTE + ENN and SMOTE + Tomek). Discrimination, calibration, and statistical uncertainty were assessed using standard metrics, including ROC AUC, precision–recall AUC, Brier score, expected calibration error, and participant-level bootstrap resampling (2000 replicates). Model interpretability was examined using SHAP analyses (DeepExplainer with GradientExplainer fallback), providing cohort-level and patient-level explanations.

Evaluation was conducted on both the natural-prevalence (imbalanced) test set and a balanced 1:1 diagnostic subset to characterise deployment-relevant behaviour and intrinsic discriminative ability, respectively, (see Section 2.6). Metric definitions follow established practice in imbalanced clinical prediction and are guided by prior work on precision–recall analysis, calibration, and model evaluation [28,29,32].

#### Balanced Test Sets

Balanced test sets are routinely used in clinical machine learning to assess a model’s intrinsic classification capability independently of outcome prevalence. By enforcing equal class representation, metrics such as recall, precision, and AUC reflect discrimination rather than prevalence-driven effects. This practice is widely adopted in high-quality medical AI studies: for example, the BOUND lung cancer prescreening model demonstrated that reduced AUPR under natural prevalence primarily reflects rarity rather than model weakness [42], while hypoglycaemia prediction studies in type 2 diabetes have shown that accuracy and PPV can be misleading under severe imbalance without complementary balanced evaluation [43]. Similar dual-evaluation strategies have been used in human–machine cooperation studies to contrast controlled and real-world performance [44]. Accordingly, balanced diagnostic sets are used here for fair model comparison, while imbalanced test sets remain essential for estimating realistic PPV, NPV, and deployment-ready clinical performance.

Interpretation ranges for all reported metrics are summarised in Table A14.

**1. Accuracy.**
  $$\mathrm{Accuracy}=\frac{TP+TN}{TP+TN+FP+FN}$$
  Measures overall correctness but is dominated by the majority (non-hypoglycaemia) class and is therefore reported only contextually.
**2. Precision (Positive Predictive Value).**
  $$\mathrm{Precision}=\frac{TP}{TP+FP}$$
  Indicates the proportion of predicted hypoglycaemic events that were correct; higher precision reflects fewer false alarms.
**3. Recall (Sensitivity, True Positive Rate).**
  $$\mathrm{Recall}=\frac{TP}{TP+FN}$$
  Represents the proportion of true hypoglycaemic events correctly identified; reflects clinical sensitivity but trades off with precision.
**4. Specificity (True Negative Rate).**
  $$\mathrm{Specificity}=\frac{TN}{TN+FP}$$
  Measures the ability to avoid false positives among non-hypoglycaemic hours.
**5. F1 Score (Harmonic Mean of Precision and Recall).**
  $$F1=2\times \frac{\mathrm{Precision}\times \mathrm{Recall}}{\mathrm{Precision}+\mathrm{Recall}}$$
  The weighted F1 ($F1_{w}$) averages class-specific F1 values according to label frequency, balancing sensitivity and precision.
**6. Area Under the ROC Curve (ROC AUC).**
  $$\mathrm{ROC}\;\mathrm{AUC}=\int_{0}^{1}TPR\left(FPR\right)\;d\left(FPR\right)$$
  Evaluates overall discrimination between positive and negative classes across thresholds; higher values indicate stronger separability.
**7. Area Under the Precision–Recall Curve (PR AUC).**
  $$\mathrm{PR}\;\mathrm{AUC}=\int_{0}^{1}\mathrm{Precision}\left(\mathrm{Recall}\right)\;d\left(\mathrm{Recall}\right)$$
  Emphasises minority-class performance and is more informative than ROC AUC for imbalanced datasets [28,29].
**8. Brier Score.**
  $$\mathrm{Brier}=\frac{1}{N}\sum_{i=1}^{N}{\left(p_{i}-y_{i}\right)}^{2}$$
  Assesses probability calibration—how close predicted probabilities are to observed outcomes; lower values denote better calibration [32].
**9. Expected Calibration Error (ECE).**
  $$\mathrm{ECE}=\sum_{m=1}^{M}\frac{|B_{m}|}{N}|\mathrm{acc}\left(B_{m}\right)-\mathrm{conf}\left(B_{m}\right)|$$
  Quantifies the mean difference between predicted and empirical accuracy across probability bins; smaller ECE implies stronger reliability [31].
**10. Youden’s *J* Statistic.**
  J=Sensitivity+Specificity−1
  Identifies the optimal threshold balancing sensitivity and specificity [32].
**11. Bootstrap Confidence Intervals.** Non-parametric bootstrap resampling (2000 replicates) was applied to derive 95% confidence intervals for AUC and F1; pairwise AUC differences were compared using the DeLong test for correlated ROC curves [33].
**12. Alert Stability (N-of-M Rule).** During rolling forecasts (1–12 h), alerts were triggered only when at least N=2 of M=3 consecutive windows exceeded the prediction threshold, suppressing transient spikes and enforcing temporally stable behaviour [35].
  Collectively, these metrics evaluate **discrimination**, **calibration**, and **robustness**, providing a comprehensive performance framework for hypoglycaemia prediction under class imbalance.

**Table A14:** Interpretation ranges and qualitative assessment for evaluation metrics, adapted from prior work on evaluation for imbalanced classification and clinical prediction [28,29,31,32,33,35].

Metric	Typical Range	Qualitative Interpretation	Desirable Direction
**Accuracy** [32]	0.50–0.70	Fair (imbalanced sensitivity limited)	↑ Higher better
	0.70–0.85	Good overall discrimination	
	>0.85	Excellent, but may mask minority errors	
**Precision (PPV)** [28,29]	<0.40	Weak, many false positives	↑ Higher better
	0.40–0.60	Moderate, balanced alert reliability	
	>0.60	Strong, clinically trustworthy alerts	
**Recall (Sensitivity)** [32]	<0.40	Low event capture	↑ Higher better
	0.40–0.60	Balanced for rare-event detection	
	>0.60	High sensitivity (good early detection)	
**Specificity** [32]	<0.80	Many false alarms	↑ Higher better
	0.80–0.95	Good alarm control	
	>0.95	Excellent false-alarm suppression	
**F1 Score (Weighted)** [28,29]	<0.70	Limited precision–recall balance	↑ Higher better
	0.70–0.85	Good trade-off	
	>0.85	Excellent, balanced performance	
**ROC AUC** [32,33]	<0.70	Poor discrimination	↑ Higher better
	0.70–0.85	Acceptable/good	
	0.85–0.90	Strong	
	>0.90	Excellent (state of the art)	
**PR AUC** [28,29]	<0.20	Weak for minority class	↑ Higher better
	0.20–0.35	Moderate improvement	
	0.35–0.50	Strong, clinically useful	
	>0.50	Excellent rare-event detection	
**Brier Score** [32]	>0.10	Poorly calibrated	↓ Lower better
	0.05–0.10	Acceptable	
	0.02–0.05	Good calibration	
	<0.02	Excellent calibration	
**ECE** [31]	>0.05	Misaligned probabilities	↓ Lower better
	0.02–0.05	Acceptable alignment	
	<0.02	Excellent calibration	
**Youden’s*****J*** [32]	<0.30	Weak threshold discrimination	↑ Higher better
	0.30–0.60	Good sensitivity–specificity balance	
	>0.60	Excellent diagnostic separation	
**Bootstrap CI width (95%)** [33]	>0.10	High uncertainty/small sample	↓ Narrower better
	0.05–0.10	Acceptable reliability	
	<0.05	Stable estimate, robust model	
**Alert Stability (N-of-M)** [35]	<70% stable alerts	Unreliable alarms	↑ Higher better
	70–85%	Balanced sensitivity/noise control	
	>85%	Excellent temporal reliability	

↑ indicates that higher values are desirable; ↓ indicates that lower values are desirable.
